# Mechanisms and Contingencies of Stress in University Students: A Systematic Scoping Review of Stress Mediators and Moderators

**DOI:** 10.3390/bs16020235

**Published:** 2026-02-07

**Authors:** Francisco Javier Mariscal-Garcia, Samuel García-Arellano, Ilce Valeria Román-Fernández, José Esael Pineda-Sánchez, Pedro Juárez-Rodríguez

**Affiliations:** 1 Instituto de Investigación en Ciencias Biomédicas, Centro Universitario de Ciencias de la Salud, Universidad de Guadalajara, Guadalajara 44340, Mexicoilce.romanfer@academicos.udg.mx (I.V.R.-F.); 2 Doctorado en Psicología de la Salud, Departamento de Psicología Básica, Centro Universitario de Ciencias de la Salud, Universidad de Guadalajara, Guadalajara 44340, Mexico; 3 Instituto de Ciencias de la Salud, Facultad de Psicología, Universidad Autónoma del Estado de Hidalgo, Pachuca 42000, Mexico; 4 Centro de Evaluación e Investigación Psicológica, Centro Universitario de Ciencias de la Salud, Universidad de Guadalajara, Guadalajara 44340, Mexico

**Keywords:** stress, mediation, moderation, conditional process analysis, university students

## Abstract

The aim of this study was to examine the contingencies involved in the stress process in university students by identifying evidence obtained through conditional process analyses. A systematic scoping review of mixed study designs was carried out following the methodological framework of Arksey and O’Malley and PRISMA-ScR guidelines. PubMed, Web of Science, Scopus, Redalyc, and Wdg. Búsqueda were searched for original studies published in full through August 2025 that reported conditional process analysis with stress as the outcome. After screening 1033 records, a total of 30 articles met the inclusion criteria. The main perspectives used in the included studies involved individuals’ beliefs about themselves and their future, highlighting the importance of personal resources and their relationship to perceived stress. The studies show a clear predominance of mediation analysis (25/30) over moderation analyses (2/30), along with limited inclusion of physiological (3/30) or multimodal measurements (1/30). Our synthesis provides a basis for advancing the understanding of the stress process in university students, indicating eight general perspectives of study to account for the intervening variables: standards for oneself, motivation, cognitive content, repetitive negative thinking, self-regulatory psychological resources, current status, social resources and environmental demands, and coping orientation.

## 1. Introduction

Stress is a natural process inherent in being alive. It can occur when environmental stimuli are perceived to be beyond one’s own capabilities, resources, or abilities; such demands are seen as a threat or demand (Lazarus, 1966; Lazarus & Folkman, 1984). Stress sets in motion a series of physiological and psychological processes to cope with the stressor (Lazarus & Cohen, 1977).

The most widely accepted theories of stress consider individual resources to be an important aspect of the stress response to a specific stimulus. These resources correspond to the individual’s personality traits, abilities, skills, capacities, previous experiences, and current state of the organism (Folkman et al., 1986). However, much less attention is paid to those factors that appear to condition individual responses to the same stressor and that act as mechanisms and/or contingencies in the relationship between stressors and the psychophysiological responses they can trigger. Moreover, some assumptions of these theories are often accepted without testing (Finseth et al., 2024), and when tested, the results are mainly based on self-reports, and only to a lesser extent on physiological outcomes or multimodal approaches (Darwish et al., 2025; Shanbhog & Medikonda, 2023).

Lazarus and Folkman’s transactional theory is widely used in various contexts because of its cognitive focus on the relationship between the person and the environment in the context of the stress response (de Cordova et al., 2024; Desrichard et al., 2018; Lazarus & Folkman, 1984; Spătaru et al., 2024). This theory defines stress as an imbalance in the perception of environmental demands and the person’s resources available to respond to those demands (Lazarus & Folkman, 1984). Within this conceptual framework, there are three moments in which an individual evaluates (1) the environmental stimulus (as a threat, harm/loss, or challenge), (2) what can be done to cope with that demand, and (3) whether those actions are efficient. In the latter case, evaluations and actions are repeated until a satisfactory outcome is reached (Lazarus, 1966).

### 1.1. Stress in University Students

The university context is particularly relevant for understanding stress, as it simultaneously concentrates multiple demands that characterize stress processes. There is evaluative pressure inherent in the teaching–learning process; high cognitive demands; increasing autonomy and responsibilities; and exposure to personal, social, and financial stressors (Ding et al., 2025; Nelson, 2021). Moreover, the nature of stressors present in the academic setting is predominantly chronic, which facilitates the analysis of stress processes, coping strategies, and their cumulative effects on well-being.

University life often coincides with a transitional period in the life cycle, known as emerging adulthood (Arnett, 2000; Nelson, 2021), characterized by change and instability. These changes can originate in different areas: biological, social, psychological, identity, and relational (Arnett, 2000). Notably, Solmi et al. (2022) highlighted that biological changes are related to the first onset of several mental disorders before age 25 in half the general population, making this life stage crucial for the health trajectory of individuals.

In the university context, academic stress has been conceptualized as a multidimensional construct that influences students’ mental health (Chacón et al., 2025). It is attributed to the intensification of educational competition, the social and academic expectations associated with higher education, excessive workload, inadequate time management skills, and fear of failure, among others (Ding et al., 2025; Iqra, 2024). In this regard, universities have implemented programmes and services to offer psychological help, focusing on alleviating students’ distress and emotional problems. Nevertheless, the demands and activities inherent to the higher education context tend to draw both time and energy from the students, reducing their likelihood of attending such services (Rodríguez-Saéz et al., 2025).

Furthermore, students are continuously exposed to stressors outside the academic sphere, the most notable of which, according to the scientific literature, are health issues, financial pressure, and interpersonal relationship problems (Wang et al., 2023). Stress is a primary risk factor for several neuropsychiatric disorders (Sanacora et al., 2022), mainly due to the chronic activation of the Hypothalamic–Pituitary–Adrenal axis and its effects on structural and functional changes in the brain, which are thought to contribute to the development, treatment resistance, and chronicity of such disorders (Dutton et al., 2022). However, the complex bilateral relationship between the biological and psychological spheres warrants further research (O’Connor et al., 2021; Ortega et al., 2024).

The complex interplay between variables can be elucidated through conditional process analysis, which examines the environmental factors that are inherent to the antecedent–outcome relationship and modify the outcome (Hayes & Rockwood, 2020; Hayes, 2022). These analyses can help explain why individuals exhibit a wide variety of responses (i.e., psychological, physiological, or behavioral) to the same stimuli and conditions.

### 1.2. Conditional Process Analysis

This type of analysis provides an inferential framework that goes beyond regression analysis, allowing scientists to answer questions such as through what process does an independent variable (antecedent) affect a dependent variable (outcome), and under what conditions is that process strengthened, weakened, or disappears? When properly conducted, it can uncover complex processes and serve as a starting point for causal inferences (Hayes & Rockwood, 2020). Reviewing the contingencies and mechanisms involved in the stress process, which alter and diversify the response to stress, can provide relevant information for a deeper understanding of the phenomenon of stress in university students.

Mediation arises when the effect of an independent variable (X) on an outcome variable (Y) is transmitted through an intervening variable, known as the mediator (M) or mechanism. In this framework, X influences M, which in turn influences Y, creating an indirect pathway in addition to any direct effect X may have on Y (Igartua & Hayes, 2021). Mediation can be classified according to the number and arrangement of mediators involved. Simple mediation involves a single mediator, while more complex models may feature multiple mediators. When two or more mediators operate independently, this is referred to as parallel mediation. Alternatively, in chain or sequential mediation, one mediator influences another, forming a series of linked mediators through which the effect of X on Y passes (Hayes, 2022).

Moderation refers to the interaction between two variables, where their combined effect influences the outcome variable (Y) without one variable directly affecting the other. In this context, the moderator (W) or contingency is not caused by the independent variable (X) and does not have a direct impact on Y. However, the presence of W can alter the relationship between X and Y, illustrating how moderators play a pivotal role in shaping the nature and strength of the relationship between variables (Hayes, 2022).

Complex models often incorporate both mediating and moderating variables. The interplay between these elements is referred to as a conditional process (Igartua & Hayes, 2021). Conditional process analyses, introduced by Hayes, represent a broad methodological category that encompasses the interactions between moderation and mediation. Since their introduction, these analyses have gained wide acceptance and use within the scientific community (Hayes, 2022). In this sense, structural equation modeling (SEM) and the PROCESS macro are two major tools for conducting contingency analysis. Both are based on regression analysis, but they differ in how they accept or reject models, leading to varying robustness and sample size needs (see Hayes et al., 2017, for details).

In terms of addressing heterogeneity in study designs, stress measures, and the conceptualization of stress in the scientific literature, the scoping review methodology is ideal, as it provides an overview of the existing literature, incorporating multiple levels of evidence that enrich knowledge, contributing to the identifying study designs, quality, and outcome measures; prioritizing areas of research; and preventing redundant studies (Khalil et al., 2025). For this reason, we undertook a scoping review to provide a broader interpretation of the mechanisms affecting the stress response, its indirect effects, and the interactions identified through conditional process analysis.

Therefore, the main aims of this review were (a) to identify the evidence obtained through conditional process analyses reported in the literature; (b) to characterize and classify the evidence according to the nature of the variables studied, stress measurements, and the type of conditional process analysis; (c) to examine the contingencies and mechanisms involved in the stress process in university students; (d) to outline future directions for research and the development of interventions aimed at reducing stress in university students.

## 2. Materials and Methods

Following Arksey and O’Malley’s (2005) methodological framework, this scoping review was carried out in five stages: (1) formulating the research question; (2) identifying relevant studies; (3) selecting studies; (4) charting the data; and (5) collating, summarizing, and reporting the findings. To ensure transparency and rigor, this review adhered to the Preferred Reporting Items for Systematic Reviews and Meta-Analyses extension for Scoping Reviews (PRISMA-ScR) guidelines (Tricco et al., 2018). No protocol was pre-registered, and since no original data were collected from human participants, ethical approval was not required. The final review was completed on 30 August 2025.

### 2.1. Research Question

What variables play an intervening role in the stress response in university students?

### 2.2. Search Strategy

To ensure a comprehensive and up-to-date synthesis, we conducted a literature search in the following databases: Web of Science, PubMed, Scopus, Redalyc, and Wdg. Búsqueda (a search engine and database developed by the Universidad de Guadalajara), focusing on peer-reviewed manuscripts published in English and Spanish.

Based on pilot searches, we identified keywords in the scientific literature using Boolean operators, and we determined the following search terms to retrieve relevant literature broadly focusing on stress mediators and moderators in university students: “stress”, “stress response”, “mediation analysis”, “moderation analysis”, and “students”. For example, the following search string was used in PubMed: (((“stress response” [Title/Abstract]) OR (‘stress’ [Title/Abstract])) AND (“mediation analysis” [Title/Abstract])) OR (“moderation analysis” [Title/Abstract]) AND (“students” [Title/Abstract]).

### 2.3. Eligibility

Studies were included that met the following inclusion criteria: (a) original research articles; (b) studies conducted with university students; (c) quantitative, non-experimental and quasi-experimental designs in which at least one conditional process analysis is performed; (d) studies investigating stress outcomes (perceived stress and physiological reactivity to stress); and (e) studies that performed at least one type of conditional process analysis (i.e., mediation, moderation, moderated mediation) based on the Causal Steps, PROCESS, and SEM procedures, in which the outcome variable was the perception of stress or the physiological response to stress. Studies that did not meet at least one of these criteria were excluded. The search was not restricted to any specific time period.

### 2.4. Study Selection

Duplicated records were identified and removed manually by the first author. Retrieved articles were screened by all the authors based on their titles and abstracts. The articles whose eligibility was unclear passed through a full-text review. The selected studies were assessed for eligibility using predefined inclusion and exclusion criteria, as well as for quality using the checklists specifically designed for survey-based research by the Center for Evidence-Based Management (CEBM, 2020) and the Quality Assessment for Survey Studies in Psychology (Protogerou & Hagger, 2020). The screening was conducted by the first and fifth authors according to the predetermined criteria for eligibility, using a double-blind approach to minimize any risk of bias, with discrepancies discussed until consensus was reached. In addition, these quality assessments were included as a descriptive measure of the studies.

To increase clarity, we explicitly distinguished between conditional process analysis performed with PROCESS and other procedures (based on SEM and on the Baron–Kenny procedure). By incorporating articles with analyses based on PROCESS, SEM, and the causal steps approach, we provide a broad and comprehensive perspective on the subject, placing the findings within the broader body of research. However, studies that measure stress as part of psychological distress—where stress is combined with measures of anxiety and depression—or that did not specifically treat stress as the primary outcome variable were excluded from the analysis.

## 3. Results

**Figure 1:**
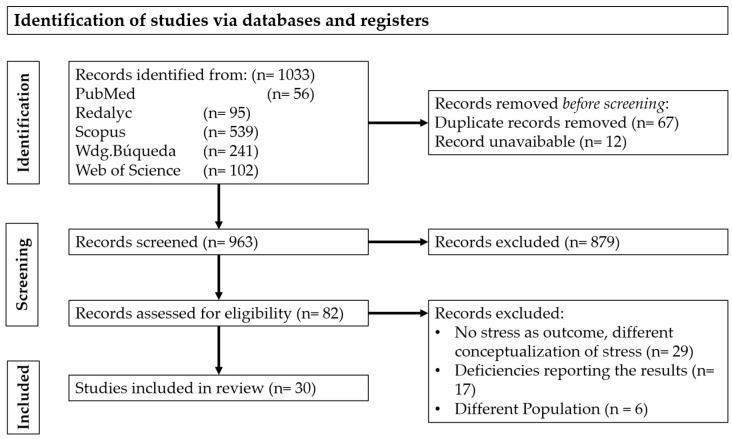
Flow chart of the selection process.

The initial database search using the predetermined terms yielded a total of 1033 records from databases. After manually removing the duplicates and the unavailable records, the number of records was reduced to 956. A total of 879 records were excluded after a thorough screening based on their titles and abstracts. This process resulted in a total of 82 articles, which were independently assessed for eligibility by two authors. Finally, 30 articles were selected and subjected to analysis for final data extraction. No articles prior to 2014 were included after the screening process as a result of the criteria used. Figure 1 outlines a detailed description of this process.

**Figure 2:**
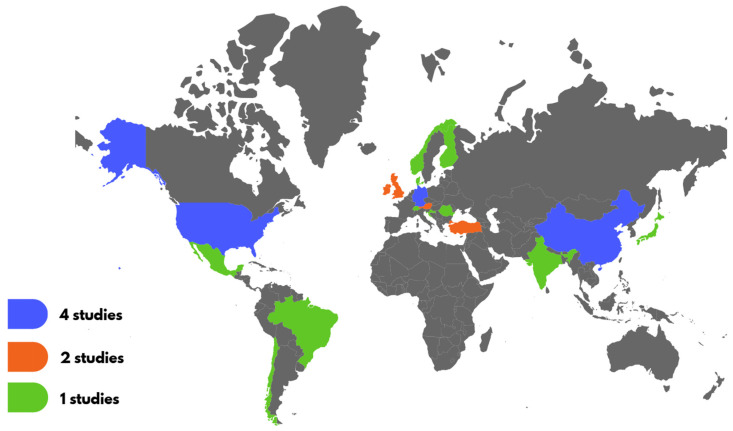
Study distribution.

The selected articles were mainly from China, Germany, and the USA (four studies each); Austria, the UK, Ireland, and Türkiye (two studies); and Austria, Brazil, Chile, Croatia, Denmark, England, Finland, India, Japan, Mexico, Norway, Romania, and Switzerland (one study). Notably, only three articles came from South America (Brazil, Chile, and Mexico), and none were from Africa or Oceania (see Figure 2).

Twenty-five of the selected articles were focused on mediation analyses, three on conditional process analyses, and two on moderation. Three studies considered physiological measures of stress reactivity, and only one included both a physiological and a self-reported measure. The quality of evidence was assessed using the CEBM checklist for critical appraisal of the quasi-experimental design studies (CEBM, 2020) and the Quality Assessment for Survey Studies in Psychology (Q-SSP) Checklist for the non-experimental design studies (Protogerou & Hagger, 2020) (see Supplementary Materials). Studies meeting half of the criteria were considered to be of satisfactory quality using the CEBM, and those meeting 70% of the criteria were considered to be of acceptable quality using the Q-SSP. Overall, 70% (21/30) of the included studies met the criteria to be considered of acceptable or satisfactory quality: 6/13 cross-sectional studies, 2/3 longitudinal studies, and 13/14 quasi-experimental studies (Table 1).

### 3.1. Cross-Sectional Studies

Cross-sectional studies can be classified into two types according to their general approach: (1) negative conditions, whose presence worsens the stress response; and (2) cognitive processes, focused on the interaction between different variables and stress. These studies assess three types of variables as conditional: coping and individual conditions, symptoms, and characteristics.

Coping and conditions: These studies included coping styles (Straud & McNaughton-Cassill, 2018) and strategies (Camilleri et al., 2022), as well as individual variables that may change even in similar situations, such as sleep quality (Garg et al., 2023), sense of control (Precht et al., 2023), self-control and resilience (Tafoya et al., 2023), and work-privacy conflicts (Efimov et al., 2024). Among these studies, only one focused on moderation (Efimov et al., 2024); notably, this study is the only one that did not find evidence supporting their hypothesis on conditional effects.

Straud and McNaughton-Cassill (2018) found an indirect effect of self-blame on stress through proactive coping, which acts as a buffer to the direct effect of self-blame on stress. In turn, Garg et al. (2023) explored a mediating effect of sleep quality on the relationship between Internet Gaming Disorder (IGD) and stress, attenuating the detrimental effects of IGD.

Tafoya et al. (2023) found a sequential buffering effect of self-control and resilience on the relationship between sleep and stress. That is, low sleep quality and duration increase stress perception, and this effect is mitigated sequentially by self-control and resilience.

During the COVID-19 pandemic, Precht et al. (2023) found that the beneficial effect of physical activity on stress was mediated by the sense of control. Another study related to the pandemic was conducted by Efimov et al. (2024), who found no significant interaction between the perceived impact of the pandemic on studies and work–life conflicts. Finally, Camilleri et al. (2022) analyzed the impact of the pandemic (measured through avoidance, hyperarousal, and intrusion) on students’ perceived stress, finding that both avoidance and hyperarousal favor dysfunctional coping, resulting in higher stress levels.

*Symptoms*: These studies focused on symptoms of disorders and other negative variables that worsen the stress response, such as insomnia symptoms (Bradford et al., 2021), dysfunctional attitudes (Odaci & Çikrikçi, 2022), and depersonalization (O’Rourke & Egan, 2023). The three studies that addressed this found statistically significant indirect effects.

Bradford et al. (2021) results suggest that individuals with an eveningness chronotype tend to have more insomnia symptoms, which increases stress. Moreover, O’Rourke and Egan (2023) examined the relationship between emotional dysregulation and stress through the role of depersonalization and insecure attachment (avoidance and anxiety), showing an exacerbating effect of attachment avoidance and attachment anxiety on depersonalization, which, in turn, increased stress. On the other hand, Odaci and Çikrikçi (2022) showed that problematic internet use increases stress indirectly through dysfunctional attitudes.

*Individual characteristics*: These studies assessed personality traits and other variables that hardly change over time, such as mindfulness traits (Lu et al., 2018), resilience (Yalcin-Siedentopf et al., 2021; Deng et al., 2023), and self-concept clarity (Ren et al., 2025). It is worth mentioning that this category wields the most complex models in these types of studies: parallel mediation (Lu et al., 2018) and conditional process analysis (Ren et al., 2025; Yalcin-Siedentopf et al., 2021).

Regarding resilience, Yalcin-Siedentopf et al. (2021) examined how perceived social support influences perceived stress through resilience and found sex-differentiated reducing effects of resilience on perceived stress. They therefore ran a second-stage conditional model with sex as a moderator of the resilience–stress relationship, highlighting a stronger effect of resilience on reducing perceived stress in women than in men. Furthermore, Deng et al. (2023) found that resilience mediates the caring ability–stress relationship.

Concerning parallel mediation, Lu et al. (2018) proposed that mindfulness traits interact with each other to reduce perceived stress and tested two models in which two different traits (acting with awareness and observing) affect perceived stress directly and indirectly through non-judgment and non-reactivity. This means that acting with awareness decreases perceived stress by increasing non-judgment, while observing has a similar effect by increasing non-reactivity.

Finally, Ren et al. (2025) tested a first-stage conditional model in which value conflicts strongly affect self-concept clarity in those with an independent self-construal, which, in turn, affects stress perception.

### 3.2. Longitudinal Studies

Among the observational studies with a longitudinal design, only two were conducted; Stevenson and Akram (2022) found that self-hatred and inadequacy mediated the relationship between baseline perfectionism and perceived stress at follow-up 15 weeks later in UK university students, providing a novel target for student-based interventions.

On the other hand, Petak and Maričić (2025) found that rumination and worry sequentially mediated the relationship between sleep quality and stress at the follow-up ~14 weeks later. Furthermore, their results provide support for a bidirectional relationship between sleep quality and stress.

Furthermore, Schönfeld et al. (2018) recorded data from students over three successive years, finding that daily stressors influence stress after two years through general self-efficacy.

It is worth mentioning that, according to Hayes (2022), longitudinal studies give greater robustness to conditional process analyses than cross-sectional studies, given that repeated measurements allow for observing variations over time in both the outcome, the antecedent, and the intervening variables, as well as to establish a causal order in their relationship based on their sequential changes.

### 3.3. Quasi-Experimental Studies

Experimental manipulation of the antecedent variable or environmental conditions allows even more robust conclusions to be drawn (Hayes, 2022), making this type of evidence of even greater value in the study of variables involved in the stress process in university students. Broadly, experimental studies seek to assess the effects of a specific intervention on perceived stress among university students. Several investigations have tested mindfulness-based interventions to determine the variables underlying their observed positive effects on students, finding a variety of mediators such as rumination, self-compassion and mindfulness (Hosseinzadeh & İl, 2022), mindfulness (Küchler et al., 2022), self-compassion and worry (Gu et al., 2018), discontinuity of mind (Juul et al., 2021), positive affect (Sousa et al., 2021), and acceptance coping (Ștefan et al., 2018); as well as moderators such as conscientiousness (De Vibe et al., 2015).

Different interventions have been tested, such as Cognitive Behavioral Therapy for insomnia (CBT-I) (Ubara et al., 2022), Sport Education for Physical Activity (Li et al., 2022), and Acceptance and Commitment Therapy (ACT) (Räsänen et al., 2020), finding mediators such as insomnia severity (Ubara et al., 2022), task orientation (Li et al., 2022), and non-reactivity and meaningfulness (Räsänen et al., 2020).

More heterogeneity in the rationale for interventions was observed in other studies. For example, the pre-experiment by Gutiérrez-Carmona et al. (2024) tested the effect of compassion-based training in reducing perceived stress. They found that increased self-compassion mediated the relationship between autonomous practice time and reduced stress.

O’Riordan et al. (2020) used a modified form of the Trier Social Stress Test (TSST) that included arithmetic and speech tasks to ascertain the mediating role of social relations in the relationship between type D personality and cardiovascular reactivity as an indicator of acute stress. Their results are consistent with the stress-buffering and social aggravation of stress hypotheses, which propose that both supportive and negative social relationships impact health outcomes by influencing stress appraisal and coping. Additionally, Yang et al. (2014) applied the TSST and found that self-esteem had an indirect effect on the heart rate stress response through need for social approval. Finally, a randomized controlled study based on self-determination theory conducted by Triebner et al. (2024) found that the inclusion of innovative concepts in teaching reduced the stress levels of medical students through improved motivation.

### 3.4. Classification

Based on data extracted from the included articles, categories were created to group the intervening variables used, thereby placing them within domains that reflect their nature. Categories based on theory used in previous research were also considered (Santee et al., 2023).

### 3.5. Standards for One-Self

This category includes variables that reflect how individuals relate to themselves, especially in times of difficulty or perceived failure. This includes negative comparisons with others and criticism over failing to meet internal, personal standards (*Self-criticism*) (Gilbert et al., 2004); perceived failure but a desire to improve oneself and desire to remove undesired aspects of the self (*Self-criticizing/attacking*) (Gilbert et al., 2004); and being open to and moved by one’s own suffering, experiencing feelings of caring and kindness toward oneself, taking an understanding and nonjudgmental attitude toward one’s inadequacies and failures, and recognizing that one’s own experience is part of the common human experience (*Self-compassion*) (Neff, 2003). The most studied variable in this category was self-compassion (4/5 studies).

### 3.6. Motivation

This category reflects those elements that guide the course of action on a daily basis, referring to engagement and persistence when the outcome is not the desired one or the event presents difficulties in the accomplishment of a task or a goal. This encompasses defining success as the task mastery and/or personal improvement, reflecting high competence and therefore subjective success (*Task orientation*) (Duda, 1989); learning-related behaviors driven by externally imposed rewards and punishments, or regulated by the internal rewards of self-esteem for success and by avoidance of anxiety, shame, or guilt for failure (*Controlled regulation of learning*) (Ryan & Deci, 2020); doing an activity for the inherent satisfaction of the activity itself (*Intrinsic motivation*) (Ryan & Deci, 2020); and the omission of behaviors, which are culturally sanctioned and approved but are improbable of occurrence due to a negative social evaluation (*Social desirability*) (Crowne & Marlowe, 1960). All variables in this category were used in one study.

### 3.7. Cognitive Content

The variables in this category reflect relatively stable cognitive representations that individuals hold about themselves, the world, and their ability to influence outcomes when faced with adversities. This includes negative thoughts and images that are difficult to control, accompanied by an attempt to mentally resolve problems with uncertain outcomes, which may include one or more negative consequences (*Worry*) (Borkovec et al., 1983); negative beliefs formed during the interaction process with the environment, developed in relation to oneself, others, and the world (*Dysfunctional attitudes*) (Beck, 2005); the extent to which individuals clearly and consistently define their self-concept (*Self-concept clarity*) (Campbell et al., 1996); self-concept clarity, even in individualistic cultures that promote independent self-concept (*Independent self-construal*) (Campbell et al., 1996); beliefs that one’s life events and conditions are controllable by one’s actions and not by luck (*Sense of control*) (Pearlin & Schooler, 1978); and the extent to which the individual perceives certain aspects of his/her life as being meaningful and valuable enough to give time, effort, and commitment to (*Meaningfulness*) (Antonovsky, 2002). Worry was the most studied variable in this category (2/6 studies).

### 3.8. Repetitive Negative Thinking

The variables in this category refer to processes associated with repetitive, passive, difficult-to-control, and negative self-referent thought content, which are not necessarily focused only on oneself. Encompasses experiences of unreality, detachment, or being an outside observer with respect to one’s thoughts, feelings, sensations, body, or actions (*Depersonalization*) (American Psychiatric Association, 2013); a fragmented or chaotic stream of thoughts when attention drifts away from the task at hand toward stimulus-independent thought (*Discontinuity of mind*) (Diaz et al., 2013); and conscious thoughts around a common theme that recur in the absence of environmental demands requiring the thoughts (*Rumination*) (Martin & Tesser, 1996). In this category, rumination was the most commonly employed variable (2/4).

### 3.9. Self-Regulatory Psychological Resources

This category includes variables reflecting capacities that enable individuals to regulate attention, emotion, and behavior in ways that support coping and well-being. These self-regulatory psychological resources may reduce stress by preventing the escalation (i.e., mindfulness) and occurrence (i.e., conscientiousness) of stressors. This category includes having receptive attention and awareness of present events and experiences (*Mindfulness traits*) (Brown et al., 2007); perceived ability to handle environmental difficulties and to respond adequately in demanding situations (*Self-efficacy*) (Bandura, 2006); mechanisms employed for the adequate management of emotions and thoughts (*Self-control*) (Rosenbaum, 1989); to stay healthy or recover easily despite adverse life events (*Resilience*) (Bonanno, 2004); as well as the propensity to follow socially prescribed norms for impulse control, to be goal-directed, to plan, and to be able to delay gratification (*Conscientiousness*) (Roberts et al., 2009). This category included the most studies in this review, and mindfulness was the most studied variable (7/11 studies), followed by resilience (3/11 studies).

### 3.10. Current Status

Studies have examined variables that account for a person’s status prior to the onset of stress, mainly characterized by the affect and the reservoir of energy available (i.e., tiredness vs. refreshment, satiety vs. hunger) to use each day, which may play a role in primary appraisal, such as the severity or symptoms related to the ability to initiate or maintain sleep (*Insomnia*) (National Library of Medicine, 2000), as well as satisfaction with the sleep experience, integrating aspects of sleep initiation, sleep maintenance, sleep quantity, and refreshment upon awakening (*Sleep quality*) (National Library of Medicine, 2021). Insomnia was the most commonly studied variable in this category (2/3 studies).

Interestingly, a recent article by Steffen and Anderson (2025) proposed a revised version of Lazarus and Folkman’s transactional model of stress and coping. These authors highlight the affective and physiological status of the individuals when facing stressful situations. They propose that both affections and physiology influence the appraisals and coping processes, indicating that the status in which individuals face stressors will affect their responses to stressors, lessening or exacerbating their interpretations of an event.

### 3.11. Social Resources and Environmental Demands

This category includes constructs that reflect how people perceive and experience their social environment, the demands of their roles, and social factors that may alleviate or exacerbate their stress perception. This includes the perception that the workload has a negative impact on private life, or vice versa (*Work-privacy conflicts*) (Garthus-Niegel et al., 2016); the interactions with others are perceived as negative, such as rejecting and attacking (*Perceived hostility/rejection*) (Cyranowski et al., 2013); the social relationships are viewed as available to provide emotional or instrumental support (*Emotional/Instrumental support*) (Cyranowski et al., 2013); and the importance attributed to one’s role in a determined groupal task (*Perceived role*) (Newton et al., 2000). The variables in this category were studied in the same proportion (once).

### 3.12. Coping Orientation

The variables in this category represent cognitive and behavioral efforts that individuals use to manage stress and challenging situations. This includes perceiving potential stressors as challenges rather than threats in an active and forward-looking manner (*proactive coping*) (Schwarzer & Taubert, 2002); coping responses that arguably are less useful (*dysfunctional coping*) (Carver et al., 1989); construing a stressful transaction in positive terms (*positive reinterpretation and growth coping*) (Carver et al., 1989); and to accept the reality of a stressful situation in circumstances in which the stressor is something that must be accommodated to (*acceptance coping*) (Carver et al., 1989). These variables were also studied in the same proportion (once) (an overview of the classification is provided in Table 2).

## 4. Discussion

The main aims of this scoping review were to examine the contingencies and mechanisms involved in the stress process among university students and to show how conditional process analysis is an appropriate approach to address questions about the mechanisms underlying the observed relationships. Our synthesis of scientific literature on the subject suggests great heterogeneity in the study of the contingencies of the stress process in university students. In this regard, we propose eight main categories to group these contingencies: standards for oneself, motivation, cognitive content, repetitive negative thoughts, self-regulatory psychological resources, current situation, social resources and environmental demands, and coping orientation.

The transactional model of stress and coping provides a useful framework for formulating and testing hypotheses about the stress process and its relation to physical and mental health (Lazarus & Folkman, 1984), emphasizing the importance of appraisal and coping as mechanisms of the ongoing relationship between the person and the environment (Lazarus, 1966; Lazarus & Folkman, 1984).

According to the original model, appraisals give rise to emotions whose quality and intensity depend on both the personal meaning attributed to the event (primary evaluation) and the perceived alternatives for coping with it (secondary evaluation) (Lazarus & Lazarus, 1994; Roseman & Smith, 2001). The situation may involve goals, values, or beliefs that have different personal meanings for each subject, or because some individuals have greater resources to influence the outcomes of that situation (Folkman, 1997). However, primary and secondary appraisal are interdependent, influencing each other as part of a single, common process (Harris, 2001).

Coping refers to the thoughts and actions people use to manage distress (emotion-focused coping), manage the problem causing the distress (problem-focused coping), and sustain positive well-being (meaning-focused coping) (Lazarus & Folkman, 1984). Further coping theory developments (Carver et al., 1989) expand the relevance of actions taken to solve the stressors or mitigate their effects in the stress process.

Generally, stress has been studied from the perspective of the individuals’ resources to cope with challenges, adversities, or demands of their context. Individuals’ interpretations of stressors and coping behaviors have been studied to a lesser extent, although they appear to be more useful in predicting stress responses (Allen et al., 2014). Additionally, little attention has been paid to the current state of individuals, whose assessment seems to be limited in use as a statistical control rather than as a possible stress contingency, as recently proposed (Steffen & Anderson, 2025). Furthermore, heterogeneity in the variables analyzed reflects the heterogeneous nature of stressors, as well as the wide diversity of experiences, which are certainly influenced by equally diverse individual factors (Santee et al., 2023).

The greater number of studies on *self-regulatory psychological resources* (11 studies) and *cognitive content* (six studies) shows a trend in the literature toward seeking individual resources that can mitigate the consequences of chronic stress. This emphasis reflects a focus on secondary appraisal (Lazarus & Folkman, 1984)—that is, on what the individual has or can do to resolve the stressful event, while neglecting variables associated with primary appraisal, as evidenced by the number of studies in the categories of *current state* (three studies) and *social resources and environmental demands* (three studies), which may also influence the multidimensional process of stress (Steffen & Anderson, 2025).

### 4.1. Limitations and Future Research Directions

Our findings may be restricted by the search strategy employed, which may have limited the results, although efforts were made to be systematic and comprehensive by searching five databases. In this sense, the scoping review is a particularly effective methodology for addressing broad research questions, allowing for the inclusion of diverse study designs and methods for conditional process analyses (Munn et al., 2018).

Although we treated them separately from their antecedent variable, these analyses should be treated with caution when attempting to extrapolate their results to populations other than the original sample. Resampling techniques perform their calculations based on data from the sample obtained, so repeating the analysis with another sample may lead to different results. Furthermore, these models must be considered in their entirety, as they reflect the relationship between the variables included in them. Nevertheless, our research focused on variables that could intervene in the perception of stress in the face of an undefined stimulus, allowing us to focus on those variables by directing our attention to them rather than to the stressors included in the models.

The results come mainly from cross-sectional studies, as well as from research that predominantly used simple conditional models of analysis. Future studies in the field should pursue longitudinal and quasi-experimental designs, as well as multimodal approaches, in order to strengthen conclusions drawn about stress contingencies and mechanisms, as well as developing more complex models where different stress contingencies and mechanisms are included. According to previous recommendations (Hayes & Rockwood, 2020; Igartua & Hayes, 2021), this would enable a comparison of contingencies and account for their interactions, providing a deeper understanding of the phenomenon of stress and what should be changed to reduce its deleterious effects on students’ health. Furthermore, future research should focus on more detailed analyses of the relationship between stressors and stress, enabling a deeper understanding of the entire phenomenon.

Furthermore, heterogeneity when analyzing and reporting results represents difficulties in integrating them, which can interfere with research interpretation, replicability, evidence synthesis, and policy application, as noted in previous reports (Protogerou & Hagger, 2020; Rizzo et al., 2022). Thus, we recommend following established guidelines for conducting and reporting mediation analyses (e.g., Lee et al., 2021; Schuler et al., 2025) in order to homogenize the reports’ structures and elements, enabling a comparison of different studies focused on conditional process analysis. This would facilitate the advancement of theories and interventions on stress through critical analysis and comparison of their impact and mechanisms.

The present scoping review provides a basis for advancing our understanding of the stress process, highlighting different targets of psychosocial interventions for health promotion and disease prevention that could address the identified characteristics to attenuate their negative effects and reinforce their protective role on stress, directing the focus not toward eradicating stress but toward empowering individuals in their stress processes, giving them tools to reduce its negative effects and to cope effectively with it. This study also demonstrates that conditional process analyses are a valuable tool for uncovering the mechanisms underlying the effects of interventions aimed at mitigating stress.

### 4.2. Conclusions

Our synthesis proposes eight general perspectives to account for intervening variables in the stress process among university students, with a clear emphasis on coping resources (secondary appraisal) and less emphasis on assessing the personal significance of the stressful event and the current state (primary appraisal). The literature also shows a preference for self-reported data, simpler analyses, and cross-sectional designs over multimodal measurements, complex models, and longitudinal approaches. Although useful, the preferred approaches yield results that should be treated with caution. This scoping review also provides a basis for advancing our understanding of the stress process among university students and highlights several targets for universities and stakeholders to develop and deliver informed psychosocial interventions.

## Figures and Tables

**Table 1:** Characteristics of the included studies.

Authors	Country	No.	Type of Analysis	Independent Variable(s)	Mediating or Moderating Variable(s)	Stress Measurement (Instrument)	CEBM/Q-SSP
**Cross-sectional**							
Straud and McNaughton-Cassill (2018)	USA	261	Mediation	Self-blame coping style	Proactive coping	DASS-21 Stress subscale	Questionable
Lu et al. (2018)	USA	99	Parallel mediation	Observing trait Acting with awareness trait	Non-Reactivity trait Non-Judging trait	PSS	Acceptable
Bradford et al. (2021)	UK	190	Mediation	Chronotype	Insomnia symptoms	DASS-21 Stress subscale	Acceptable
Yalcin-Siedentopf et al. (2021)	Austria	503	Second-stage conditional process	Perceived social support	M: Resilience W: Sex	PSS	Questionable
Camilleri et al. (2022)	USA	676	Parallel Mediation	Impact of the COVID-19 pandemic	Dysfunctional Coping	DASS-21 Stress subscale	Acceptable
Odaci and Çikrikçi (2022)	Türkiye	543	Mediation	Problematic Internet Use	Dysfunctional Attitudes	DASS-21 Stress subscale	Questionable
Deng et al. (2023)	China	295	Mediation	Caring ability	Resilience	PSS	Questionable
Garg et al. (2023)	India	795	Mediation	Internet Gaming Disorder	Sleep Quality	PSS	Questionable
O’Rourke and Egan (2023)	Ireland	313	Mediation	Insecure Attachment	Depersonalization	DASS-21 Stress subscale	Questionable
Precht et al. (2023)	Germany	568	Mediation	Physical Activity Frequency	Sense of control	DASS-21 Stress subscale	Questionable
Tafoya et al. (2023)	Mexico	32	Chain Mediation	Sleep Quality and Duration	Resilience Self-control	PSS	Acceptable
Efimov et al. (2024)	Germany	342	Moderation	Perceived impact of COVID-19 pandemic	Work–privacy conflict	PSS	Acceptable
Ren et al. (2025)	China	752	First-stage conditional process	Value conflict	M: Self-Concept clarity W: independent self-construal	DASS-21 Stress subscale	Acceptable
**Longitudinal studies**							
Schönfeld et al. (2018)	China	2160	Mediation	Daily stressors	General Self-Efficacy	DASS-21 Stress subscale	Questionable
Stevenson and Akram (2022)	UK	220 baseline/84 follow-up	Parallel mediation	Self-oriented perfectionism Socially prescribed perfectionism	Self-Criticizing/Attacking	PSS	Acceptable
Petak and Maričić (2025)	Croatia	302	Chain mediation	Sleep quality	Rumination Worry	DASS-21 Stress subscale	Acceptable
**Quasi-experimental studies**							
Yang et al. (2014)	China	41	Mediation	Self-esteem	Social desirability	HR	Satisfactory
De Vibe et al. (2015)	Norway	288	Moderation	MBSR intervention	Mindfulness Conscientiousness	Perceived Medical School Stress	Satisfactory
Gu et al. (2018)	UK	120	Mediation	Mindfulness-Based Self-Help Intervention	Mindfulness Self-compassion Trait worry	PSS	Satisfactory
Ștefan et al. (2018)	Romania	46	Mediation	MBSR Intervention	Self-Compassion Positive reinterpretation and Growth Coping Acceptance Coping	PSS	Satisfactory
O’Riordan et al. (2020)	Ireland	195	Parallel mediation	Type D personality	Emotional Support Instrumental Support Perceived Rejection Perceived Hostility	SBP, DBP, HR, CO, TPR	Satisfactory
Räsänen et al. (2020)	Finland	68	Mediation	ACT-based Intervention	Meaningfulness Non-Reactivity trait	PSS	Satisfactory
Juul et al. (2021)	Denmark	67	Mediation	MBSR Intervention	Discontinuity of Mind	PSS	Satisfactory
Sousa et al. (2021)	Bazil	40	Second-stage conditional process	Mindfulness Training	M: Mindfulness state W: Mindfulness trait	PSS Plasma Cortisol	Satisfactory
Hosseinzadeh and İl (2022)	Türkiye	101	Parallel mediation	MBCT-based Intervention	Rumination Self-Compassion Mindfulness	DASS-21 Stress subscale	Satisfactory
Küchler et al. (2022)	Germany, Switzerland and Austria	149	Mediation	Mindfulness-based Intervention	Mindfulness	PSQ	Satisfactory
Li et al. (2022)	USA	236	Mediation	Sport Education Sessions	Perceived Role Task Orientation	PSS	Satisfactory
Ubara et al. (2022)	Japan	41	Mediation	CBT for insomnia	Insomnia severity	DASS-21 Stress subscale	Satisfactory
Gutiérrez-Carmona et al. (2024)	Chile	45	Mediation	Autonomous self-compassion and mindfulness practice	Self-compassion	PSS	Questionable
Triebner et al. (2024)	Germany	105	Parallel mediation	Selfdetermination-based practical course	Intrinsic motivation Controlled regulation of learning	VAS	Satisfactory

Note: The models presented were simplified when multiple outcomes were present. For critical appraisal using CEBM, studies meeting half of the criteria (12) were considered Satisfactory. For critical appraisal using the Q-SSP, a score of 70% was considered Acceptable. M: Mediator; W: Moderator; PSS: Perceived Stress Scale; VAS: Visual Analogue Scale; DASS: Depression, Anxiety, Stress Scale; PSQ: Perceived Stress Questionnaire; CEBM: Centre for Evidence-Based Medicine; Q-SSP: Quality of Survey Studies in Psychology Checklist; SBP: Systolic Blood Pressure; DBP: Diastolic Blood Pressure; HR: Heart Rate; CO: Cardiac Output; TPR: Total Peripheral Resistance; MBSR: Mindfulness-Based Stress Reduction; CBT: Cognitive Behavioral Therapy; MBCT: Mindfulness-Based Cognitive Therapy.

**Table 2:** Classification of the studies according to the extracted categories.

Category	Variables	Analysis	Design	Authors
**Standards for oneself**	Self-Criticizing/Attacking	Parallel Mediation	Longitudinal	Stevenson and Akram (2022)
	Self-Compassion	Mediation	Quasiexperimental	Gu et al. (2018)
	Self-Compassion	Parallel Mediation	Quasiexperimental	Hosseinzadeh and İl (2022)
	Self-Compassion	Mediation	Quasiexperimental	Gutiérrez-Carmona et al. (2024)
	Self-Compassion	Mediation	Quasiexperimental	Ștefan et al. (2018)
**Motivation**	Social desirability	Mediation	Quasiexperimental	Yang et al. (2014)
	Task Orientation	Mediation	Quasiexperimental	Li et al. (2022)
	Controlled regulation of learning	Parallel mediation	Quasiexperimental	Triebner et al. (2024)
**Cognitive content**	Trait worry	Mediation	Quasiexperimental	Gu et al. (2018)
	Dysfunctional Attitudes	Mediation	Cross-sectional	Odaci and Çikrikçi (2022)
	Meaningfulness	Mediation	Quasiexperimental	Räsänen et al. (2020)
	Worry	Chain Mediation	Longitudinal	Petak and Maričić (2025)
	Sense of control	Mediation	Cross-sectional	Precht et al. (2023)
	Self-Concept clarity Independent self-construal	First-stage conditional process	Cross-sectional	Ren et al. (2025)
**Repetitive negative thinking**	Rumination	Parallel mediation	Quasiexperimental	Hosseinzadeh and İl (2022)
	Depersonalization	Mediation	Cross-sectional	O’Rourke and Egan (2023)
	Rumination	Chain Mediation	Longitudinal	Petak and Maričić (2025)
	Discontinuity of Mind	Mediation	Quasiexperimental	Juul et al. (2021)
**Self-regulatory psychological resources**	Mindfulness	Mediation	Quasiexperimental	Gu et al. (2018)
	Mindfulness	Parallel Mediation	Quasiexperimental	Hosseinzadeh and İl (2022)
	Non-Reactivity trait Non-Judging trait	Parallel Mediation	Cross-sectional	Lu et al. (2018)
	Resilience	Second-stage conditional process	Cross-sectional	Yalcin-Siedentopf et al. (2021)
	Resilience	Mediation	Cross-sectional	Deng et al. (2023)
	Resilience Self-control	Chain Mediation	Cross-sectional	Tafoya et al. (2023)
	General Self-Efficacy	Mediation	Longitudinal	Schönfeld et al. (2018)
	Mindfulness Conscientiousness	Moderation	Quasiexperimental	De Vibe et al. (2015)
	Non-Reactivity trait	Mediation	Quasiexperimental	Räsänen et al. (2020)
	Mindfulness state Mindfulness trait	Second-stage conditional process	Quasiexperimental	Sousa et al. (2021)
	Mindfulness	Mediation	Quasiexperimental	Küchler et al. (2022)
**Current status**	Insomnia Symptoms	Mediation	Cross-sectional	Bradford et al. (2021)
	Sleep Quality	Mediation	Cross-sectional	Garg et al. (2023)
	Insomnia Severity	Mediation	Quasiexperimental	Ubara et al. (2022)
**Coping orientation**	Proactive coping	Mediation	Cross-sectional	Straud and McNaughton-Cassill (2018)
	Dysfunctional Coping	Parallel mediation	Cross-sectional	Camilleri et al. (2022)
	Positive reinterpretation and Growth Coping Acceptance Coping	Mediation	Quasiexperimental	Ștefan et al. (2018)
**Social resources and environmental demands**	Perceived Role	Mediation	Quasiexperimental	Li et al. (2022)
	Work–privacy conflict	Moderation	Cross-sectional	Efimov et al. (2024)
	Emotional Support Instrumental Support Perceived Rejection Perceived Hostility	Parallel Mediation	Quasiexperimental	O’Riordan et al. (2020)

Note: Some studies measured variables from different categories; hence, some appear in more than one category.

## Data Availability

No new data were created or analyzed in this study. Data sharing is not applicable to this article.

## Supplementary Materials

The following supporting information can be downloaded at: https://www.mdpi.com/article/10.3390/bs16020235/s1, Quality assessments of the included studies.
