# Supplementary material for: Mechanisms and Contingencies of Stress in University Students: A Systematic Scoping Review of Stress Mediators and Moderators
Source: Behav Sci (Basel). 2026 Feb 7;16(2):235. doi: 10.3390/bs16020235 (PMC12938083; doi:10.3390/bs16020235)
Supplement: Supplementary file 1 [file behavsci-16-00235-s001.zip › behavsci-4064056-supplementary.pdf]

CEBMa center for Evidence-Based Management

Critical Appraisal of a Controlled Study

Study: Does personality moderate the effects of mindfulness training for medical and psychology students?

Appraisal questions	Yes	Can't tell	No
1. *Did the study address a clearly focused question / issue?*	X		
2. *Is the research method (study design) appropriate for answering the research question?*	X		
3. *Were there enough subjects (employees, teams, divisions, organizations) in the study to establish that the findings did not occur by chance?*	X		
4. *Were subjects randomly allocated to the experimental and control group? If not, could this have introduced bias?*	X		
5. *Are objective inclusion / exclusion criteria used?*	X		
6. *Were both groups comparable at the start of the study?*	X		
7. *Were objective and unbiased outcome criteria used?*			X
8. *Are objective and validated measurement methods used to measure the outcome? If not, was the outcome assessed by someone who was unaware of the group assignment (i.e. was the assessor blinded)?*	X		
9. *Is the size effect practically relevant?*	X		
10. *How precise is the estimate of the effect? Were confidence intervals given?*			X
11. *Could there be confounding factors that haven't been accounted for?*	X		
12. *Can the results be applied to your organization?*	X		

Adapted from Crombie, *The Pocket Guide to Critical Appraisal*; the critical appraisal approach used by the Oxford Centre for Evidence Medicine, checklists of the Dutch Cochrane Centre, BMJ editor's checklists and the checklists of the EPPI Centre.

Cite as: Center for Evidence Based Management (July, 2014), Critical Appraisal Checklist for a Controlled Study. Retrieved (month, day, year) from https://www.cebma.org

Critical Appraisal of a Controlled Study

Study: Investigating the specific effects of an online mindfulness-based self-help intervention on stress and underlying mechanisms

Appraisal questions	Yes	Can't tell	No
1. *Did the study address a clearly focused question / issue?*	X		
2. *Is the research method (study design) appropriate for answering the research question?*	X		
3. *Were there enough subjects (employees, teams, divisions, organizations) in the study to establish that the findings did not occur by chance?*	X		
4. *Were subjects randomly allocated to the experimental and control group? If not, could this have introduced bias?*	X		
5. *Are objective inclusion / exclusion criteria used?*		X	
6. *Were both groups comparable at the start of the study?*	X		
7. *Were objective and unbiased outcome criteria used?*			X
8. *Are objective and validated measurement methods used to measure the outcome? If not, was the outcome assessed by someone who was unaware of the group assignment (i.e. was the assessor blinded)?*	X		
9. *Is the size effect practically relevant?*		X	
10. *How precise is the estimate of the effect? Were confidence intervals given?*	X		
11. *Could there be confounding factors that haven't been accounted for?*	X		
12. *Can the results be applied to your organization?*	X		

Adapted from Crombie, *The Pocket Guide to Critical Appraisal*; the critical appraisal approach used by the Oxford Centre for Evidence Medicine, checklists of the Dutch Cochrane Centre, BMJ editor's checklists and the checklists of the EPPI Centre.

Cite as: Center for Evidence Based Management (July, 2014), Critical Appraisal Checklist for a Controlled Study. Retrieved (month, day, year) from https://www.cebma.org

Critical Appraisal of a Controlled Study

Study: Effectiveness of compassion training on stress and anxiety: A pre-experimental study on nursing students

Appraisal questions	Yes	Can't tell	No
1. *Did the study address a clearly focused question / issue?*	X		
2. *Is the research method (study design) appropriate for answering the research question?*	X		
3. *Were there enough subjects (employees, teams, divisions, organizations) in the study to establish that the findings did not occur by chance?*	X		
4. *Were subjects randomly allocated to the experimental and control group? If not, could this have introduced bias?*			X
5. *Are objective inclusion / exclusion criteria used?*	X		
6. *Were both groups comparable at the start of the study?*		X	
7. *Were objective and unbiased outcome criteria used?*			X
8. *Are objective and validated measurement methods used to measure the outcome? If not, was the outcome assessed by someone who was unaware of the group assignment (i.e. was the assessor blinded)?*	X		
9. *Is the size effect practically relevant?*		X	
10. *How precise is the estimate of the effect? Were confidence intervals given?*			X
11. *Could there be confounding factors that haven't been accounted for?*	X		
12. *Can the results be applied to your organization?*	X		

Adapted from Crombie, *The Pocket Guide to Critical Appraisal*; the critical appraisal approach used by the Oxford Centre for Evidence Medicine, checklists of the Dutch Cochrane Centre, BMJ editor's checklists and the checklists of the EPPI Centre.

Cite as: Center for Evidence Based Management (July, 2014), Critical Appraisal Checklist for a Controlled Study. Retrieved (month, day, year) from https://www.cebma.org

CEBMa
center for
Evidence-Based Management

Critical Appraisal of a Controlled Study

Study: The effectiveness of a brief mindfulness-based program for social work students in two separate modules: Traditional and online

Appraisal questions	Yes	Can't tell	No
1. *Did the study address a clearly focused question / issue?*	X		
2. *Is the research method (study design) appropriate for answering the research question?*	X		
3. *Were there enough subjects (employees, teams, divisions, organizations) in the study to establish that the findings did not occur by chance?*	X		
4. *Were subjects randomly allocated to the experimental and control group? If not, could this have introduced bias?*	X		
5. *Are objective inclusion / exclusion criteria used?*	X		
6. *Were both groups comparable at the start of the study?*	X		
7. *Were objective and unbiased outcome criteria used?*			X
8. *Are objective and validated measurement methods used to measure the outcome? If not, was the outcome assessed by someone who was unaware of the group assignment (i.e. was the assessor blinded)?*	X		
9. *Is the size effect practically relevant?*		X	
10. *How precise is the estimate of the effect? Were confidence intervals given?*	X		
11. *Could there be confounding factors that haven't been accounted for?*	X		
12. *Can the results be applied to your organization?*	X		

Adapted from Crombie, *The Pocket Guide to Critical Appraisal*; the critical appraisal approach used by the Oxford Centre for Evidence Medicine, checklists of the Dutch Cochrane Centre, BMJ editor's checklists and the checklists of the EPPI Centre.

Cite as: Center for Evidence Based Management (July, 2014), Critical Appraisal Checklist for a Controlled Study. Retrieved (month, day, year) from https://www.cebma.org

CEBMa
center for
Evidence-Based Management

Critical Appraisal of a Controlled Study

Study: The effects of a mindfulness program on mental health in students at an undergraduate program for teacher education: A randomized controlled trial in real-life

Appraisal questions	Yes	Can't tell	No
1. *Did the study address a clearly focused question / issue?*	X		
2. *Is the research method (study design) appropriate for answering the research question?*	X		
3. *Were there enough subjects (employees, teams, divisions, organizations) in the study to establish that the findings did not occur by chance?*	X		
4. *Were subjects randomly allocated to the experimental and control group? If not, could this have introduced bias?*	X		
5. *Are objective inclusion / exclusion criteria used?*	X		
6. *Were both groups comparable at the start of the study?*	X		
7. *Were objective and unbiased outcome criteria used?*			X
8. *Are objective and validated measurement methods used to measure the outcome? If not, was the outcome assessed by someone who was unaware of the group assignment (i.e. was the assessor blinded)?*	X		
9. *Is the size effect practically relevant?*		X	
10. *How precise is the estimate of the effect? Were confidence intervals given?*	X		
11. *Could there be confounding factors that haven't been accounted for?*	X		
12. *Can the results be applied to your organization?*	X		

Adapted from Crombie, *The Pocket Guide to Critical Appraisal*; the critical appraisal approach used by the Oxford Centre for Evidence Medicine, checklists of the Dutch Cochrane Centre, BMJ editor's checklists and the checklists of the EPPI Centre.

Cite as: Center for Evidence Based Management (July, 2014), Critical Appraisal Checklist for a Controlled Study. Retrieved (month, day, year) from https://www.cebma.org

Critical Appraisal of a Controlled Study

Study: Effectiveness, acceptability, and mechanisms of change of the internet-based intervention StudiCare mindfulness for college students: A randomized controlled trial

Appraisal questions	Yes	Can't tell	No
1. *Did the study address a clearly focused question / issue?*	X		
2. *Is the research method (study design) appropriate for answering the research question?*	X		
3. *Were there enough subjects (employees, teams, divisions, organizations) in the study to establish that the findings did not occur by chance?*			X
4. *Were subjects randomly allocated to the experimental and control group? If not, could this have introduced bias?*	X		
5. *Are objective inclusion / exclusion criteria used?*	X		
6. *Were both groups comparable at the start of the study?*	X		
7. *Were objective and unbiased outcome criteria used?*			X
8. *Are objective and validated measurement methods used to measure the outcome? If not, was the outcome assessed by someone who was unaware of the group assignment (i.e. was the assessor blinded)?*	X		
9. *Is the size effect practically relevant?*		X	
10. *How precise is the estimate of the effect? Were confidence intervals given?*	X		
11. *Could there be confounding factors that haven't been accounted for?*	X		
12. *Can the results be applied to your organization?*	X		

Adapted from Crombie, *The Pocket Guide to Critical Appraisal*; the critical appraisal approach used by the Oxford Centre for Evidence Medicine, checklists of the Dutch Cochrane Centre, BMJ editor's checklists and the checklists of the EPPI Centre.

Cite as: Center for Evidence Based Management (July, 2014), Critical Appraisal Checklist for a Controlled Study. Retrieved (month, day, year) from https://www.cebma.org

Critical Appraisal of a Controlled Study

Study: Targeting well-being and physical activity through sport education in higher education

Appraisal questions	Yes	Can't tell	No
1. *Did the study address a clearly focused question / issue?*	X		
2. *Is the research method (study design) appropriate for answering the research question?*	X		
3. *Were there enough subjects (employees, teams, divisions, organizations) in the study to establish that the findings did not occur by chance?*	X		
4. *Were subjects randomly allocated to the experimental and control group? If not, could this have introduced bias?*	X		
5. *Are objective inclusion / exclusion criteria used?*	X		
6. *Were both groups comparable at the start of the study?*	X		
7. *Were objective and unbiased outcome criteria used?*			X
8. *Are objective and validated measurement methods used to measure the outcome? If not, was the outcome assessed by someone who was unaware of the group assignment (i.e. was the assessor blinded)?*	X		
9. *Is the size effect practically relevant?*	X		
10. *How precise is the estimate of the effect? Were confidence intervals given?*	X		
11. *Could there be confounding factors that haven't been accounted for?*	X		
12. *Can the results be applied to your organization?*	X		

Adapted from Crombie, *The Pocket Guide to Critical Appraisal*; the critical appraisal approach used by the Oxford Centre for Evidence Medicine, checklists of the Dutch Cochrane Centre, BMJ editor's checklists and the checklists of the EPPI Centre.

Cite as: Center for Evidence Based Management (July, 2014), Critical Appraisal Checklist for a Controlled Study. Retrieved (month, day, year) from https://www.cebma.org

CEBMa center for Evidence-Based Management

Critical Appraisal of a Controlled Study

Study: Type D personality and cardiovascular reactivity to acute stress: The mediating effects of social support and negative social relationships

Appraisal questions	Yes	Can't tell	No
1. *Did the study address a clearly focused question / issue?*	X		
2. *Is the research method (study design) appropriate for answering the research question?*	X		
3. *Were there enough subjects (employees, teams, divisions, organizations) in the study to establish that the findings did not occur by chance?*	X		
4. *Were subjects randomly allocated to the experimental and control group? If not, could this have introduced bias?*	X		
5. *Are objective inclusion / exclusion criteria used?*	X		
6. *Were both groups comparable at the start of the study?*	X		
7. *Were objective and unbiased outcome criteria used?*	X		
8. *Are objective and validated measurement methods used to measure the outcome? If not, was the outcome assessed by someone who was unaware of the group assignment (i.e. was the assessor blinded)?*	X		
9. *Is the size effect practically relevant?*		X	
10. *How precise is the estimate of the effect? Were confidence intervals given?*			X
11. *Could there be confounding factors that haven't been accounted for?*	X		
12. *Can the results be applied to your organization?*	X		

Adapted from Crombie, *The Pocket Guide to Critical Appraisal*; the critical appraisal approach used by the Oxford Centre for Evidence Medicine, checklists of the Dutch Cochrane Centre, BMJ editor's checklists and the checklists of the EPPI Centre.

Cite as: Center for Evidence Based Management (July, 2014), Critical Appraisal Checklist for a Controlled Study. Retrieved (month, day, year) from https://www.cebma.org

CEBMa center for Evidence-Based Management

Critical Appraisal of a Controlled Study

Study: Examining mediators of change in wellbeing, stress, and depression in a blended, Internet-based, ACT intervention for university students

Appraisal questions	Yes	Can't tell	No
1. *Did the study address a clearly focused question / issue?*	X		
2. *Is the research method (study design) appropriate for answering the research question?*	X		
3. *Were there enough subjects (employees, teams, divisions, organizations) in the study to establish that the findings did not occur by chance?*	X		
4. *Were subjects randomly allocated to the experimental and control group? If not, could this have introduced bias?*	X		
5. *Are objective inclusion / exclusion criteria used?*	X		
6. *Were both groups comparable at the start of the study?*	X		
7. *Were objective and unbiased outcome criteria used?*			X
8. *Are objective and validated measurement methods used to measure the outcome? If not, was the outcome assessed by someone who was unaware of the group assignment (i.e. was the assessor blinded)?*	X		
9. *Is the size effect practically relevant?*		X	
10. *How precise is the estimate of the effect? Were confidence intervals given?*	X		
11. *Could there be confounding factors that haven't been accounted for?*	X		
12. *Can the results be applied to your organization?*	X		

Adapted from Crombie, *The Pocket Guide to Critical Appraisal*; the critical appraisal approach used by the Oxford Centre for Evidence Medicine, checklists of the Dutch Cochrane Centre, BMJ editor's checklists and the checklists of the EPPI Centre.

Cite as: Center for Evidence Based Management (July, 2014), Critical Appraisal Checklist for a Controlled Study. Retrieved (month, day, year) from https://www.cebma.org

CEBMa center for Evidence-Based Management

Critical Appraisal of a Controlled Study

Study: Brief mindfulness-based training and mindfulness trait attenuate psychological stress in university students: A randomized controlled trial

Appraisal questions	Yes	Can't tell	No
1. *Did the study address a clearly focused question / issue?*	X		
2. *Is the research method (study design) appropriate for answering the research question?*	X		
3. *Were there enough subjects (employees, teams, divisions, organizations) in the study to establish that the findings did not occur by chance?*	X		
4. *Were subjects randomly allocated to the experimental and control group? If not, could this have introduced bias?*	X		
5. *Are objective inclusion / exclusion criteria used?*	X		
6. *Were both groups comparable at the start of the study?*	X		
7. *Were objective and unbiased outcome criteria used?*	X		
8. *Are objective and validated measurement methods used to measure the outcome? If not, was the outcome assessed by someone who was unaware of the group assignment (i.e. was the assessor blinded)?*	X		
9. *Is the size effect practically relevant?*		X	
10. *How precise is the estimate of the effect? Were confidence intervals given?*	X		
11. *Could there be confounding factors that haven't been accounted for?*	X		
12. *Can the results be applied to your organization?*	X		

Adapted from Crombie, *The Pocket Guide to Critical Appraisal*; the critical appraisal approach used by the Oxford Centre for Evidence Medicine, checklists of the Dutch Cochrane Centre, BMJ editor's checklists and the checklists of the EPPI Centre.

Cite as: Center for Evidence Based Management (July, 2014), Critical Appraisal Checklist for a Controlled Study. Retrieved (month, day, year) from https://www.cebma.org

CEBMa center for Evidence-Based Management

Critical Appraisal of a Controlled Study

Study: Investigating effects and mechanisms of a mindfulness-based stress reduction intervention in a sample of college students at risk for social anxiety

Appraisal questions	Yes	Can't tell	No
1. *Did the study address a clearly focused question / issue?*	X		
2. *Is the research method (study design) appropriate for answering the research question?*	X		
3. *Were there enough subjects (employees, teams, divisions, organizations) in the study to establish that the findings did not occur by chance?*	X		
4. *Were subjects randomly allocated to the experimental and control group? If not, could this have introduced bias?*	X		
5. *Are objective inclusion / exclusion criteria used?*	X		
6. *Were both groups comparable at the start of the study?*	X		
7. *Were objective and unbiased outcome criteria used?*			X
8. *Are objective and validated measurement methods used to measure the outcome? If not, was the outcome assessed by someone who was unaware of the group assignment (i.e. was the assessor blinded)?*	X		
9. *Is the size effect practically relevant?*	X		
10. *How precise is the estimate of the effect? Were confidence intervals given?*	X		
11. *Could there be confounding factors that haven't been accounted for?*	X		
12. *Can the results be applied to your organization?*	X		

Adapted from Crombie, *The Pocket Guide to Critical Appraisal*; the critical appraisal approach used by the Oxford Centre for Evidence Medicine, checklists of the Dutch Cochrane Centre, BMJ editor's checklists and the checklists of the EPPI Centre.

Cite as: Center for Evidence Based Management (July, 2014), Critical Appraisal Checklist for a Controlled Study. Retrieved (month, day, year) from https://www.cebma.org

Critical Appraisal of a Controlled Study

Study: Promoting motivation and reducing stress in medical students by utilizing self-determination theory—A randomized controlled trial in practical psychiatry courses

Appraisal questions	Yes	Can't tell	No
1. *Did the study address a clearly focused question / issue?*	X		
2. *Is the research method (study design) appropriate for answering the research question?*	X		
3. *Were there enough subjects (employees, teams, divisions, organizations) in the study to establish that the findings did not occur by chance?*	X		
4. *Were subjects randomly allocated to the experimental and control group? If not, could this have introduced bias?*	X		
5. *Are objective inclusion / exclusion criteria used?*			X
6. *Were both groups comparable at the start of the study?*	X		
7. *Were objective and unbiased outcome criteria used?*			X
8. *Are objective and validated measurement methods used to measure the outcome? If not, was the outcome assessed by someone who was unaware of the group assignment (i.e. was the assessor blinded)?*	X		
9. *Is the size effect practically relevant?*		X	
10. *How precise is the estimate of the effect? Were confidence intervals given?*	X		
11. *Could there be confounding factors that haven't been accounted for?*	X		
12. *Can the results be applied to your organization?*	X		

Adapted from Crombie, *The Pocket Guide to Critical Appraisal*; the critical appraisal approach used by the Oxford Centre for Evidence Medicine, checklists of the Dutch Cochrane Centre, BMJ editor's checklists and the checklists of the EPPI Centre.

Cite as: Center for Evidence Based Management (July, 2014), Critical Appraisal Checklist for a Controlled Study. Retrieved (month, day, year) from https://www.cebma.org

Critical Appraisal of a Controlled Study

Study: How does e-mail-delivered cognitive behavioral therapy work for young adults (18–28 years) with Insomnia? Mediators of changes in insomnia, depression, anxiety, and stress

Appraisal questions	Yes	Can't tell	No
1. *Did the study address a clearly focused question / issue?*	X		
2. *Is the research method (study design) appropriate for answering the research question?*	X		
3. *Were there enough subjects (employees, teams, divisions, organizations) in the study to establish that the findings did not occur by chance?*			X
4. *Were subjects randomly allocated to the experimental and control group? If not, could this have introduced bias?*	X		
5. *Are objective inclusion / exclusion criteria used?*	X		
6. *Were both groups comparable at the start of the study?*		X	
7. *Were objective and unbiased outcome criteria used?*			X
8. *Are objective and validated measurement methods used to measure the outcome? If not, was the outcome assessed by someone who was unaware of the group assignment (i.e. was the assessor blinded)?*	X		
9. *Is the size effect practically relevant?*		X	
10. *How precise is the estimate of the effect? Were confidence intervals given?*	X		
11. *Could there be confounding factors that haven't been accounted for?*	X		
12. *Can the results be applied to your organization?*	X		

Adapted from Crombie, *The Pocket Guide to Critical Appraisal*; the critical appraisal approach used by the Oxford Centre for Evidence Medicine, checklists of the Dutch Cochrane Centre, BMJ editor's checklists and the checklists of the EPPI Centre.

Cite as: Center for Evidence Based Management (July, 2014), Critical Appraisal Checklist for a Controlled Study. Retrieved (month, day, year) from https://www.cebma.org

CEBMa
center for
Evidence-Based Management

Critical Appraisal of a Controlled Study

Study: Correlation between self-esteem and stress response in Chinese college students: The mediating role of the need for social approval

Appraisal questions	Yes	Can't tell	No
1. *Did the study address a clearly focused question / issue?*	X		
2. *Is the research method (study design) appropriate for answering the research question?*	X		
3. *Were there enough subjects (employees, teams, divisions, organizations) in the study to establish that the findings did not occur by chance?*			X
4. *Were subjects randomly allocated to the experimental and control group? If not, could this have introduced bias?*	X		
5. *Are objective inclusion / exclusion criteria used?*	X		
6. *Were both groups comparable at the start of the study?*	X		
7. *Were objective and unbiased outcome criteria used?*			X
8. *Are objective and validated measurement methods used to measure the outcome? If not, was the outcome assessed by someone who was unaware of the group assignment (i.e. was the assessor blinded)?*	X		
9. *Is the size effect practically relevant?*		X	
10. *How precise is the estimate of the effect? Were confidence intervals given?*	X		
11. *Could there be confounding factors that haven't been accounted for?*	X		
12. *Can the results be applied to your organization?*	X		

Adapted from Crombie, *The Pocket Guide to Critical Appraisal*; the critical appraisal approach used by the Oxford Centre for Evidence Medicine, checklists of the Dutch Cochrane Centre, BMJ editor's checklists and the checklists of the EPPI Centre.

Cite as: Center for Evidence Based Management (July, 2014), Critical Appraisal Checklist for a Controlled Study. Retrieved (month, day, year) from https://www.cebma.org

Quality Assessment Checklist for Survey Studies in Psychology (Q-SSP) and Guide

Study: Insomnia symptoms mediate the association between eveningness and suicidal ideation, defeat, entrapment, and psychological distress in students					
**Research domain**	**Quality item**	**Yes**	**No**	**Not stated clearly**	**N/A**
Introduction (Rationale)	1. Was the problem or phenomenon under investigation defined, described, and justified?	X			
Introduction (Rationale)	2. Was the population under investigation defined, described, and justified?	X			
Introduction (Rationale)	3. Were specific research questions and/or hypotheses stated?	X			
Introduction (Variables)	4. Were operational definitions of all study variables provided?	X			
Participants (Sampling)	5. Were participant inclusion criteria stated?	X			
Participants (Sampling)	6. Was the participant recruitment strategy described?	X			
Participants (Sampling)	7. Was a justification/ rationale for the sample size provided?		X		
Data (Collection)	8. Was the attrition rate provided? (applies to cross-sectional and prospective studies)	X			
Data (Analyses)	9. Was a method of treating attrition provided? (applies to cross-sectional and prospective studies)	X			
Data (Analyses)	10. Were the data analysis techniques justified (i.e., was the link between hypotheses/ aims / research questions and data analyses explained)?	X			
Data (Measures)	11. Were the measures provided in the report (or in a supplement) in full?		X		
Data (Measures)	12. Was evidence provided for the validity of all the measures (or instrument) used?	X			
Data (Collection)	13. Was information provided about the person(s) who collected the data (e.g., training, expertise, other demographic characteristics)?		X		
Data (Collection)	14. Was information provided about the context (e.g., place) of data collection?	X			
Data (Collection)	15. Was information provided about the duration (or start and end date) of data collection?	X			
Data (Results)	16. Was the study sample described in terms of key demographic characteristics?		X		

Data (Discussion)	17. Was discussion of findings confined to the population from which the sample was drawn?		X		
Ethics	18. Were participants asked to provide (informed) consent or assent?	X			
Ethics	19. Were participants debriefed at the end of data collection?	X			
Ethics	20. Were funding sources or conflicts of interest disclosed?	X			

SCORING (optional)

Overall Quality Score (%): 75

Compute an overall study quality score expressed as a percentage by dividing YES (Y) scores by the Total (T) number of **APPLICABLE** items and multiplying by 100. If a report fails to attain a Y score for 5 of the items, then it may be classed as of questionable quality.
Specifically:
When (T) = 20, then a score of Y/T ≥ 75% may be considered ceptable quality.
When (T) = 19, then a score of Y/T ≥ 73% may be considered ceptable quality.
When (T) = 18, then a score of Y/T ≥ 72% may be considered ceptable quality.
When (T) = 17, then a score of Y/T ≥ 70% may be considered ceptable quality.
If Y/T <75% or < 73% or < 72%, or < 70% (depending on number of applicable items), then study is of questionable quality.

Domain Quality Scores
Express domain quality scores as a simple ratio of the (Y) items, divided by the (T) applicable items.

(4 items) Introduction (Rationale/Variables) score: 4/4

(3 items) Participants (Sampling/Recruitment) score: 2/3

(10 items) Data (Collection/Analyses/Measures/Results/Discussion) score: 6/10

(3 items) Ethics score: 3/3

IN A DATA FILE, ASSIGN **1** FOR YES SCORES; **0** FOR NO OR NOT STATED CLEARLY; AND **2** = FOR NOT APPLICABLE.

Quality Assessment Checklist for Survey Studies in Psychology (Q-SSP) and Guide

Study: The Impact of COVID-19 and Associated Interventions on Mental Health: A Cross-Sectional Study in a Sample of University Students

Research domain	Quality item	Yes	No	Not stated clearly	N/A
Introduction (Rationale)	1. Was the problem or phenomenon under investigation defined, described, and justified?	X			
Introduction (Rationale)	2. Was the population under investigation defined, described, and justified?	X			
Introduction (Rationale)	3. Were specific research questions and/or hypotheses stated?	X			
Introduction (Variables)	4. Were operational definitions of all study variables provided?	X			
Participants (Sampling)	5. Were participant inclusion criteria stated?	X			
Participants (Sampling)	6. Was the participant recruitment strategy described?	X			
Participants (Sampling)	7. Was a justification/ rationale for the sample size provided?	X			
Data (Collection)	8. Was the attrition rate provided? (applies to cross-sectional and prospective studies)	X			
Data (Analyses)	9. Was a method of treating attrition provided? (applies to cross-sectional and prospective studies)		X		
Data (Analyses)	10. Were the data analysis techniques justified (i.e., was the link between hypotheses/ aims / research questions and data analyses explained)?	X			
Data (Measures)	11. Were the measures provided in the report (or in a supplement) in full?		X		
Data (Measures)	12. Was evidence provided for the validity of all the measures (or instrument) used?		X		
Data (Collection)	13. Was information provided about the person(s) who collected the data (e.g., training, expertise, other demographic characteristics)?		X		
Data (Collection)	14. Was information provided about the context (e.g., place) of data collection?		X		
Data (Collection)	15. Was information provided about the duration (or start and end date) of data collection?	X			
Data (Results)	16. Was the study sample described in terms of key demographic characteristics?	X			

Data (Discussion)	17. Was discussion of findings confined to the population from which the sample was drawn?	X			
Ethics	18. Were participants asked to provide (informed) consent or assent?	X			
Ethics	19. Were participants debriefed at the end of data collection?		X		
Ethics	20. Were funding sources or conflicts of interest disclosed?	X			

SCORING (optional)

Overall Quality Score (%): 70%

Compute an overall study quality score expressed as a percentage by dividing YES (Y) scores by the Total (T) number of **APPLICABLE** items and multiplying by 100. If a report fails to attain a Y score for 5 of the items, then it may be classed as of questionable quality.
Specifically:
When (T) = 20, then a score of Y/T ≥ 75% may be considered ceptable quality.
When (T) = 19, then a score of Y/T ≥ 73% may be considered ceptable quality.
When (T) = 18, then a score of Y/T ≥ 72% may be considered ceptable quality.
When (T) = 17, then a score of Y/T ≥ 70% may be considered ceptable quality.
If Y/T <75% or < 73% or < 72%, or < 70% (depending on number of applicable items), then study is of questionable quality.

Domain Quality Scores
Express domain quality scores as a simple ratio of the (Y) items, divided by the (T) applicable items.

(4 items) Introduction (Rationale/Variables) score: /4

(3 items) Participants (Sampling/Recruitment) score: /3

(10 items) Data (Collection/Analyses/Measures/Results/Discussion) score: /10

(3 items) Ethics score: /3

IN A DATA FILE, ASSIGN **1** FOR YES SCORES; **0** FOR NO OR NOT STATED CLEARLY; AND **2** = FOR NOT APPLICABLE.

Quality Assessment Checklist for Survey Studies in Psychology (Q-SSP) and Guide

Study: The Effect of Caring Ability on Perceived Stress Mediated by Resilience

Research domain	Quality item	Yes	No	Not stated clearly	N/A
Introduction (Rationale)	1. Was the problem or phenomenon under investigation defined, described, and justified?	X			
Introduction (Rationale)	2. Was the population under investigation defined, described, and justified?	X			
Introduction (Rationale)	3. Were specific research questions and/or hypotheses stated?	X			
Introduction (Variables)	4. Were operational definitions of all study variables provided?	X			
Participants (Sampling)	5. Were participant inclusion criteria stated?		X		
Participants (Sampling)	6. Was the participant recruitment strategy described?		X		
Participants (Sampling)	7. Was a justification/ rationale for the sample size provided?		X		
Data (Collection)	8. Was the attrition rate provided? (applies to cross-sectional and prospective studies)		X		
Data (Analyses)	9. Was a method of treating attrition provided? (applies to cross-sectional and prospective studies)		X		
Data (Analyses)	10. Were the data analysis techniques justified (i.e., was the link between hypotheses/ aims / research questions and data analyses explained)?	X			
Data (Measures)	11. Were the measures provided in the report (or in a supplement) in full?		X		
Data (Measures)	12. Was evidence provided for the validity of all the measures (or instrument) used?	X			
Data (Collection)	13. Was information provided about the person(s) who collected the data (e.g., training, expertise, other demographic characteristics)?		X		
Data (Collection)	14. Was information provided about the context (e.g., place) of data collection?		X		
Data (Collection)	15. Was information provided about the duration (or start and end date) of data collection?		X		
Data (Results)	16. Was the study sample described in terms of key demographic characteristics?		X		

Data (Discussion)	17. Was discussion of findings confined to the population from which the sample was drawn?	X			
Ethics	18. Were participants asked to provide (informed) consent or assent?	X			
Ethics	19. Were participants debriefed at the end of data collection?		X		
Ethics	20. Were funding sources or conflicts of interest disclosed?	X			

SCORING (optional)

Overall Quality Score (%): 45%

Compute an overall study quality score expressed as a percentage by dividing YES (Y) scores by the Total (T) number of **APPLICABLE** items and multiplying by 100. If a report fails to attain a Y score for 5 of the items, then it may be classed as of questionable quality.

Specifically:

When (T) = 20, then a score of Y/T ≥ 75% may be considered ceptable quality.

When (T) = 19, then a score of Y/T ≥ 73% may be considered ceptable quality.

When (T) = 18, then a score of Y/T ≥ 72% may be considered ceptable quality.

When (T) = 17, then a score of Y/T ≥ 70% may be considered ceptable quality.

If Y/T <75% or < 73% or < 72%, or < 70% (depending on number of applicable items), then study is of questionable quality.

Domain Quality Scores

Express domain quality scores as a simple ratio of the (Y) items, divided by the (T) applicable items.

(4 items) Introduction (Rationale/Variables) score: /4

(3 items) Participants (Sampling/Recruitment) score: /3

(10 items) Data (Collection/Analyses/Measures/Results/Discussion) score: /10

(3 items) Ethics score: /3

IN A DATA FILE, ASSIGN **1** FOR YES SCORES; **0** FOR NO OR NOT STATED CLEARLY; AND **2** = FOR NOT APPLICABLE.

Quality Assessment Checklist for Survey Studies in Psychology (Q-SSP) and Guide

Study: Stress perception, coping behaviors and work-privacy conflict of student midwives in times of COVID-19 pandemic: the “Healthy MidStudents” study in Germany

Research domain	Quality item	Yes	No	Not stated clearly	N/A
Introduction (Rationale)	1. Was the problem or phenomenon under investigation defined, described, and justified?	X			
Introduction (Rationale)	2. Was the population under investigation defined, described, and justified?	X			
Introduction (Rationale)	3. Were specific research questions and/or hypotheses stated?	X			
Introduction (Variables)	4. Were operational definitions of all study variables provided?	X			
Participants (Sampling)	5. Were participant inclusion criteria stated?	X			
Participants (Sampling)	6. Was the participant recruitment strategy described?	X			
Participants (Sampling)	7. Was a justification/ rationale for the sample size provided?		X		
Data (Collection)	8. Was the attrition rate provided?<br>(applies to cross-sectional and prospective studies)	X			
Data (Analyses)	9. Was a method of treating attrition provided?<br>(applies to cross-sectional and prospective studies)	X			
Data (Analyses)	10. Were the data analysis techniques justified (i.e., was the link between hypotheses/ aims / research questions and data analyses explained)?	X			
Data (Measures)	11. Were the measures provided in the report (or in a supplement) in full?		X		
Data (Measures)	12. Was evidence provided for the validity of all the measures (or instrument) used?	X			
Data (Collection)	13. Was information provided about the person(s) who collected the data (e.g., training, expertise, other demographic characteristics)?		X		
Data (Collection)	14. Was information provided about the context (e.g., place) of data collection?	X			
Data (Collection)	15. Was information provided about the duration (or start and end date) of data collection?	X			
Data (Results)	16. Was the study sample described in terms of key demographic characteristics?	X			

Data (Discussion)	17. Was discussion of findings confined to the population from which the sample was drawn?	X			
Ethics	18. Were participants asked to provide (informed) consent or assent?	X			
Ethics	19. Were participants debriefed at the end of data collection?		X		
Ethics	20. Were funding sources or conflicts of interest disclosed?	X			

SCORING (optional)

Overall Quality Score (%): 80%

Compute an overall study quality score expressed as a percentage by dividing YES (Y) scores by the Total (T) number of **APPLICABLE** items and multiplying by 100. If a report fails to attain a Y score for 5 of the items, then it may be classed as of questionable quality.

Specifically:

When (T) = 20, then a score of Y/T ≥ 75% may be considered ceptable quality.

When (T) = 19, then a score of Y/T ≥ 73% may be considered ceptable quality.

When (T) = 18, then a score of Y/T ≥ 72% may be considered ceptable quality.

When (T) = 17, then a score of Y/T ≥ 70% may be considered ceptable quality.

If Y/T <75% or < 73% or < 72%, or < 70% (depending on number of applicable items), then study is of questionable quality.

Domain Quality Scores

Express domain quality scores as a simple ratio of the (Y) items, divided by the (T) applicable items.

(4 items) Introduction (Rationale/Variables) score: /4

(3 items) Participants (Sampling/Recruitment) score: /3

(10 items) Data (Collection/Analyses/Measures/Results/Discussion) score: /10

(3 items) Ethics score: /3

IN A DATA FILE, ASSIGN **1** FOR YES SCORES; **0** FOR NO OR NOT STATED CLEARLY; AND **2** = FOR NOT APPLICABLE.

Quality Assessment Checklist for Survey Studies in Psychology (Q-SSP) and Guide

Study: The mediating role of sleep quality on the relationship between internet gaming disorder and perceived stress and suicidal behaviour among Indian medical students

Research domain	Quality item	Yes	No	Not stated clearly	N/A
Introduction (Rationale)	1. Was the problem or phenomenon under investigation defined, described, and justified?	X			
Introduction (Rationale)	2. Was the population under investigation defined, described, and justified?	X			
Introduction (Rationale)	3. Were specific research questions and/or hypotheses stated?	X			
Introduction (Variables)	4. Were operational definitions of all study variables provided?		X		
Participants (Sampling)	5. Were participant inclusion criteria stated?	X			
Participants (Sampling)	6. Was the participant recruitment strategy described?	X			
Participants (Sampling)	7. Was a justification/ rationale for the sample size provided?	X			
Data (Collection)	8. Was the attrition rate provided? (applies to cross-sectional and prospective studies)		X		
Data (Analyses)	9. Was a method of treating attrition provided? (applies to cross-sectional and prospective studies)		X		
Data (Analyses)	10. Were the data analysis techniques justified (i.e., was the link between hypotheses/ aims / research questions and data analyses explained)?	X			
Data (Measures)	11. Were the measures provided in the report (or in a supplement) in full?		X		
Data (Measures)	12. Was evidence provided for the validity of all the measures (or instrument) used?	X			
Data (Collection)	13. Was information provided about the person(s) who collected the data (e.g., training, expertise, other demographic characteristics)?		X		
Data (Collection)	14. Was information provided about the context (e.g., place) of data collection?	X			
Data (Collection)	15. Was information provided about the duration (or start and end date) of data collection?	X			
Data (Results)	16. Was the study sample described in terms of key demographic characteristics?	X			

Data (Discussion)	17. Was discussion of findings confined to the population from which the sample was drawn?	X			
Ethics	18. Were participants asked to provide (informed) consent or assent?	X			
Ethics	19. Were participants debriefed at the end of data collection?	X			
Ethics	20. Were funding sources or conflicts of interest disclosed?	X			

SCORING (optional)

Overall Quality Score (%): 45%

Compute an overall study quality score expressed as a percentage by dividing YES (Y) scores by the Total (T) number of **APPLICABLE** items and multiplying by 100. If a report fails to attain a Y score for 5 of the items, then it may be classed as of questionable quality.
Specifically:
When (T) = 20, then a score of Y/T ≥ 75% may be considered ceptable quality.
When (T) = 19, then a score of Y/T ≥ 73% may be considered ceptable quality.
When (T) = 18, then a score of Y/T ≥ 72% may be considered ceptable quality.
When (T) = 17, then a score of Y/T ≥ 70% may be considered ceptable quality.
If Y/T <75% or < 73% or < 72%, or < 70% (depending on number of applicable items), then study is of questionable quality.

Domain Quality Scores
Express domain quality scores as a simple ratio of the (Y) items, divided by the (T) applicable items.

(4 items) Introduction (Rationale/Variables) score: /4

(3 items) Participants (Sampling/Recruitment) score: /3

(10 items) Data (Collection/Analyses/Measures/Results/Discussion) score: /10

(3 items) Ethics score: /3

IN A DATA FILE, ASSIGN **1** FOR YES SCORES; **0** FOR NO OR NOT STATED CLEARLY; AND **2** = FOR NOT APPLICABLE.

Quality Assessment Checklist for Survey Studies in Psychology (Q-SSP) and Guide

Study: Self-blame and stress in undergraduate college students: The mediating role of proactive coping

Research domain	Quality item	Yes	No	Not stated clearly	N/A
Introduction (Rationale)	1. Was the problem or phenomenon under investigation defined, described, and justified?	X			
Introduction (Rationale)	2. Was the population under investigation defined, described, and justified?	X			
Introduction (Rationale)	3. Were specific research questions and/or hypotheses stated?	X			
Introduction (Variables)	4. Were operational definitions of all study variables provided?	X			
Participants (Sampling)	5. Were participant inclusion criteria stated?	X			
Participants (Sampling)	6. Was the participant recruitment strategy described?	X			
Participants (Sampling)	7. Was a justification/ rationale for the sample size provided?		X		
Data (Collection)	8. Was the attrition rate provided?<br>(applies to cross-sectional and prospective studies)	X			
Data (Analyses)	9. Was a method of treating attrition provided?<br>(applies to cross-sectional and prospective studies)		X		
Data (Analyses)	10. Were the data analysis techniques justified (i.e., was the link between hypotheses/ aims / research questions and data analyses explained)?	X			
Data (Measures)	11. Were the measures provided in the report (or in a supplement) in full?		X		
Data (Measures)	12. Was evidence provided for the validity of all the measures (or instrument) used?	X			
Data (Collection)	13. Was information provided about the person(s) who collected the data (e.g., training, expertise, other demographic characteristics)?		X		
Data (Collection)	14. Was information provided about the context (e.g., place) of data collection?			X	
Data (Collection)	15. Was information provided about the duration (or start and end date) of data collection?	X			
Data (Results)	16. Was the study sample described in terms of key demographic characteristics?	X			

Data (Discussion)	17. Was discussion of findings confined to the population from which the sample was drawn?	X			
Ethics	18. Were participants asked to provide (informed) consent or assent?	X			
Ethics	19. Were participants debriefed at the end of data collection?		X		
Ethics	20. Were funding sources or conflicts of interest disclosed?	X			

SCORING (optional)

Overall Quality Score (%): 70

Compute an overall study quality score expressed as a percentage by dividing YES (Y) scores by the Total (T) number of **APPLICABLE** items and multiplying by 100. If a report fails to attain a Y score for 5 of the items, then it may be classed as of questionable quality.
Specifically:
When (T) = 20, then a score of Y/T ≥ 75% may be considered ceptable quality.
When (T) = 19, then a score of Y/T ≥ 73% may be considered ceptable quality.
When (T) = 18, then a score of Y/T ≥ 72% may be considered ceptable quality.
When (T) = 17, then a score of Y/T ≥ 70% may be considered ceptable quality.
If Y/T <75% or < 73% or < 72%, or < 70% (depending on number of applicable items), then study is of questionable quality.

Domain Quality Scores
Express domain quality scores as a simple ratio of the (Y) items, divided by the (T) applicable items.

(4 items) Introduction (Rationale/Variables) score: /4

(3 items) Participants (Sampling/Recruitment) score: /3

(10 items) Data (Collection/Analyses/Measures/Results/Discussion) score: /10

(3 items) Ethics score: /3

IN A DATA FILE, ASSIGN **1** FOR YES SCORES; **0** FOR NO OR NOT STATED CLEARLY; AND **2** = FOR NOT APPLICABLE.

Quality Assessment Checklist for Survey Studies in Psychology (Q-SSP) and Guide

Study: The Effects of Emotion Regulation on Physical and Psychological Wellbeing in University Students: The Role of Depersonalization and Attachment Style

Research domain	Quality item	Yes	No	Not stated clearly	N/A
Introduction (Rationale)	1. Was the problem or phenomenon under investigation defined, described, and justified?	X			
Introduction (Rationale)	2. Was the population under investigation defined, described, and justified?	X			
Introduction (Rationale)	3. Were specific research questions and/or hypotheses stated?	X			
Introduction (Variables)	4. Were operational definitions of all study variables provided?	X			
Participants (Sampling)	5. Were participant inclusion criteria stated?		X		
Participants (Sampling)	6. Was the participant recruitment strategy described?	X			
Participants (Sampling)	7. Was a justification/ rationale for the sample size provided?	X			
Data (Collection)	8. Was the attrition rate provided? (applies to cross-sectional and prospective studies)		X		
Data (Analyses)	9. Was a method of treating attrition provided? (applies to cross-sectional and prospective studies)		X		
Data (Analyses)	10. Were the data analysis techniques justified (i.e., was the link between hypotheses/ aims / research questions and data analyses explained)?	X			
Data (Measures)	11. Were the measures provided in the report (or in a supplement) in full?		X		
Data (Measures)	12. Was evidence provided for the validity of all the measures (or instrument) used?	X			
Data (Collection)	13. Was information provided about the person(s) who collected the data (e.g., training, expertise, other demographic characteristics)?		X		
Data (Collection)	14. Was information provided about the context (e.g., place) of data collection?		X		
Data (Collection)	15. Was information provided about the duration (or start and end date) of data collection?		X		
Data (Results)	16. Was the study sample described in terms of key demographic characteristics?	X			

Data (Discussion)	17. Was discussion of findings confined to the population from which the sample was drawn?	X			
Ethics	18. Were participants asked to provide (informed) consent or assent?	X			
Ethics	19. Were participants debriefed at the end of data collection?	X			
Ethics	20. Were funding sources or conflicts of interest disclosed?	X			

SCORING (optional)

Overall Quality Score (%): 65%

Compute an overall study quality score expressed as a percentage by dividing YES (Y) scores by the Total (T) number of **APPLICABLE** items and multiplying by 100. If a report fails to attain a Y score for 5 of the items, then it may be classed as of questionable quality.

Specifically:

When (T) = 20, then a score of Y/T ≥ 75% may be considered ceptable quality.

When (T) = 19, then a score of Y/T ≥ 73% may be considered ceptable quality.

When (T) = 18, then a score of Y/T ≥ 72% may be considered ceptable quality.

When (T) = 17, then a score of Y/T ≥ 70% may be considered ceptable quality.

If Y/T <75% or < 73% or < 72%, or < 70% (depending on number of applicable items), then study is of questionable quality.

Domain Quality Scores

Express domain quality scores as a simple ratio of the (Y) items, divided by the (T) applicable items.

(4 items) Introduction (Rationale/Variables) score: /4

(3 items) Participants (Sampling/Recruitment) score: /3

(10 items) Data (Collection/Analyses/Measures/Results/Discussion) score: /10

(3 items) Ethics score: /3

IN A DATA FILE, ASSIGN **1** FOR YES SCORES; **0** FOR NO OR NOT STATED CLEARLY; AND **2** = FOR NOT APPLICABLE.

Quality Assessment Checklist for Survey Studies in Psychology (Q-SSP) and Guide

Study: Dysfunctional Attitudes as a Mediator in the Association Between Problematic Internet Use and Depression, Anxiety, and Stress					
**Research domain**	**Quality item**	**Yes**	**No**	**Not stated clearly**	**N/A**
Introduction (Rationale)	1. Was the problem or phenomenon under investigation defined, described, and justified?	X			
Introduction (Rationale)	2. Was the population under investigation defined, described, and justified?	X			
Introduction (Rationale)	3. Were specific research questions and/or hypotheses stated?	X			
Introduction (Variables)	4. Were operational definitions of all study variables provided?		X		
Participants (Sampling)	5. Were participant inclusion criteria stated?		X		
Participants (Sampling)	6. Was the participant recruitment strategy described?		X		
Participants (Sampling)	7. Was a justification/ rationale for the sample size provided?		X		
Data (Collection)	8. Was the attrition rate provided?<br>(applies to cross-sectional and prospective studies)		X		
Data (Analyses)	9. Was a method of treating attrition provided?<br>(applies to cross-sectional and prospective studies)		X		
Data (Analyses)	10. Were the data analysis techniques justified (i.e., was the link between hypotheses/ aims / research questions and data analyses explained)?	X			
Data (Measures)	11. Were the measures provided in the report (or in a supplement) in full?		X		
Data (Measures)	12. Was evidence provided for the validity of all the measures (or instrument) used?	X			
Data (Collection)	13. Was information provided about the person(s) who collected the data (e.g., training, expertise, other demographic characteristics)?		X		
Data (Collection)	14. Was information provided about the context (e.g., place) of data collection?	X			
Data (Collection)	15. Was information provided about the duration (or start and end date) of data collection?		X		
Data (Results)	16. Was the study sample described in terms of key demographic characteristics?	X			

Data (Discussion)	17. Was discussion of findings confined to the population from which the sample was drawn?	X			
Ethics	18. Were participants asked to provide (informed) consent or assent?	X			
Ethics	19. Were participants debriefed at the end of data collection?			X	
Ethics	20. Were funding sources or conflicts of interest disclosed?	X			

SCORING (optional)

Overall Quality Score (%): 50%

Compute an overall study quality score expressed as a percentage by dividing YES (Y) scores by the Total (T) number of **APPLICABLE** items and multiplying by 100. If a report fails to attain a Y score for 5 of the items, then it may be classed as of questionable quality.

Specifically:

When (T) = 20, then a score of Y/T ≥ 75% may be considered ceptable quality.

When (T) = 19, then a score of Y/T ≥ 73% may be considered ceptable quality.

When (T) = 18, then a score of Y/T ≥ 72% may be considered ceptable quality.

When (T) = 17, then a score of Y/T ≥ 70% may be considered ceptable quality.

If Y/T <75% or < 73% or < 72%, or < 70% (depending on number of applicable items), then study is of questionable quality.

Domain Quality Scores

Express domain quality scores as a simple ratio of the (Y) items, divided by the (T) applicable items.

(4 items) Introduction (Rationale/Variables) score: /4

(3 items) Participants (Sampling/Recruitment) score: /3

(10 items) Data (Collection/Analyses/Measures/Results/Discussion) score: /10

(3 items) Ethics score: /3

IN A DATA FILE, ASSIGN **1** FOR YES SCORES; **0** FOR NO OR NOT STATED CLEARLY; AND **2** = FOR NOT APPLICABLE.

Quality Assessment Checklist for Survey Studies in Psychology (Q-SSP) and Guide

Study: The Role of Rumination and Worry in the Bidirectional Relationship Between Stress and Sleep Quality in Students					
**Research domain**	**Quality item**	**Yes**	**No**	**Not stated clearly**	**N/A**
Introduction (Rationale)	1. Was the problem or phenomenon under investigation defined, described, and justified?	X			
Introduction (Rationale)	2. Was the population under investigation defined, described, and justified?	X			
Introduction (Rationale)	3. Were specific research questions and/or hypotheses stated?	X			
Introduction (Variables)	4. Were operational definitions of all study variables provided?	X			
Participants (Sampling)	5. Were participant inclusion criteria stated?	X			
Participants (Sampling)	6. Was the participant recruitment strategy described?	X			
Participants (Sampling)	7. Was a justification/ rationale for the sample size provided?	X			
Data (Collection)	8. Was the attrition rate provided? (applies to cross-sectional and prospective studies)	X			
Data (Analyses)	9. Was a method of treating attrition provided? (applies to cross-sectional and prospective studies)		X		
Data (Analyses)	10. Were the data analysis techniques justified (i.e., was the link between hypotheses/ aims / research questions and data analyses explained)?	X			
Data (Measures)	11. Were the measures provided in the report (or in a supplement) in full?		X		
Data (Measures)	12. Was evidence provided for the validity of all the measures (or instrument) used?	X			
Data (Collection)	13. Was information provided about the person(s) who collected the data (e.g., training, expertise, other demographic characteristics)?		X		
Data (Collection)	14. Was information provided about the context (e.g., place) of data collection?		X		
Data (Collection)	15. Was information provided about the duration (or start and end date) of data collection?	X			
Data (Results)	16. Was the study sample described in terms of key demographic characteristics?	X			

Data (Discussion)	17. Was discussion of findings confined to the population from which the sample was drawn?	X			
Ethics	18. Were participants asked to provide (informed) consent or assent?	X			
Ethics	19. Were participants debriefed at the end of data collection?		X		
Ethics	20. Were funding sources or conflicts of interest disclosed?	X			

SCORING (optional)

Overall Quality Score (%): 75%

Compute an overall study quality score expressed as a percentage by dividing YES (Y) scores by the Total (T) number of **APPLICABLE** items and multiplying by 100. If a report fails to attain a Y score for 5 of the items, then it may be classed as of questionable quality.
Specifically:
When (T) = 20, then a score of Y/T ≥ 75% may be considered ceptable quality.
When (T) = 19, then a score of Y/T ≥ 73% may be considered ceptable quality.
When (T) = 18, then a score of Y/T ≥ 72% may be considered ceptable quality.
When (T) = 17, then a score of Y/T ≥ 70% may be considered ceptable quality.
If Y/T <75% or < 73% or < 72%, or < 70% (depending on number of applicable items), then study is of questionable quality.

Domain Quality Scores
Express domain quality scores as a simple ratio of the (Y) items, divided by the (T) applicable items.

(4 items) Introduction (Rationale/Variables) score: /4

(3 items) Participants (Sampling/Recruitment) score: /3

(10 items) Data (Collection/Analyses/Measures/Results/Discussion) score: /10

(3 items) Ethics score: /3

IN A DATA FILE, ASSIGN **1** FOR YES SCORES; **0** FOR NO OR NOT STATED CLEARLY; AND **2** = FOR NOT APPLICABLE.

Quality Assessment Checklist for Survey Studies in Psychology (Q-SSP) and Guide

Study: It's all about control: Sense of control mediates the Relationship between physical activity and mental health during the COVID-19 pandemic in Germany

Research domain	Quality item	Yes	No	Not stated clearly	N/A
Introduction (Rationale)	1. Was the problem or phenomenon under investigation defined, described, and justified?	X			
Introduction (Rationale)	2. Was the population under investigation defined, described, and justified?	X			
Introduction (Rationale)	3. Were specific research questions and/or hypotheses stated?	X			
Introduction (Variables)	4. Were operational definitions of all study variables provided?	X			
Participants (Sampling)	5. Were participant inclusion criteria stated?	X			
Participants (Sampling)	6. Was the participant recruitment strategy described?	X			
Participants (Sampling)	7. Was a justification/ rationale for the sample size provided?	X			
Data (Collection)	8. Was the attrition rate provided? (applies to cross-sectional and prospective studies)		X		
Data (Analyses)	9. Was a method of treating attrition provided? (applies to cross-sectional and prospective studies)		X		
Data (Analyses)	10. Were the data analysis techniques justified (i.e., was the link between hypotheses/ aims / research questions and data analyses explained)?	X			
Data (Measures)	11. Were the measures provided in the report (or in a supplement) in full?		X		
Data (Measures)	12. Was evidence provided for the validity of all the measures (or instrument) used?		X		
Data (Collection)	13. Was information provided about the person(s) who collected the data (e.g., training, expertise, other demographic characteristics)?		X		
Data (Collection)	14. Was information provided about the context (e.g., place) of data collection?		X		
Data (Collection)	15. Was information provided about the duration (or start and end date) of data collection?	X			
Data (Results)	16. Was the study sample described in terms of key demographic characteristics?		X		

Data (Discussion)	17. Was discussion of findings confined to the population from which the sample was drawn?	X			
Ethics	18. Were participants asked to provide (informed) consent or assent?		X		
Ethics	19. Were participants debriefed at the end of data collection?		X		
Ethics	20. Were funding sources or conflicts of interest disclosed?	X			

SCORING (optional)

Overall Quality Score (%): 55%

Compute an overall study quality score expressed as a percentage by dividing YES (Y) scores by the Total (T) number of **APPLICABLE** items and multiplying by 100. If a report fails to attain a Y score for 5 of the items, then it may be classed as of questionable quality.
Specifically:
When (T) = 20, then a score of Y/T ≥ 75% may be considered ceptable quality.
When (T) = 19, then a score of Y/T ≥ 73% may be considered ceptable quality.
When (T) = 18, then a score of Y/T ≥ 72% may be considered ceptable quality.
When (T) = 17, then a score of Y/T ≥ 70% may be considered ceptable quality.
If Y/T <75% or < 73% or < 72%, or < 70% (depending on number of applicable items), then study is of questionable quality.

Domain Quality Scores
Express domain quality scores as a simple ratio of the (Y) items, divided by the (T) applicable items.

(4 items) Introduction (Rationale/Variables) score: /4

(3 items) Participants (Sampling/Recruitment) score: /3

(10 items) Data (Collection/Analyses/Measures/Results/Discussion) score: /10

(3 items) Ethics score: /3

IN A DATA FILE, ASSIGN **1** FOR YES SCORES; **0** FOR NO OR NOT STATED CLEARLY; AND **2** = FOR NOT APPLICABLE.

Quality Assessment Checklist for Survey Studies in Psychology (Q-SSP) and Guide

Study: The Effects of Value Conflicts on Stress in Chinese College Students: A Moderated Mediation Model					
**Research domain**	**Quality item**	**Yes**	**No**	**Not stated clearly**	**N/A**
Introduction (Rationale)	1. Was the problem or phenomenon under investigation defined, described, and justified?	X			
Introduction (Rationale)	2. Was the population under investigation defined, described, and justified?	X			
Introduction (Rationale)	3. Were specific research questions and/or hypotheses stated?	X			
Introduction (Variables)	4. Were operational definitions of all study variables provided?	X			
Participants (Sampling)	5. Were participant inclusion criteria stated?	X			
Participants (Sampling)	6. Was the participant recruitment strategy described?	X			
Participants (Sampling)	7. Was a justification/ rationale for the sample size provided?	X			
Data (Collection)	8. Was the attrition rate provided? (applies to cross-sectional and prospective studies)	X			
Data (Analyses)	9. Was a method of treating attrition provided? (applies to cross-sectional and prospective studies)	X			
Data (Analyses)	10. Were the data analysis techniques justified (i.e., was the link between hypotheses/ aims / research questions and data analyses explained)?	X			
Data (Measures)	11. Were the measures provided in the report (or in a supplement) in full?		X		
Data (Measures)	12. Was evidence provided for the validity of all the measures (or instrument) used?	X			
Data (Collection)	13. Was information provided about the person(s) who collected the data (e.g., training, expertise, other demographic characteristics)?		X		
Data (Collection)	14. Was information provided about the context (e.g., place) of data collection?		X		
Data (Collection)	15. Was information provided about the duration (or start and end date) of data collection?		X		
Data (Results)	16. Was the study sample described in terms of key demographic characteristics?		X		

Data (Discussion)	17. Was discussion of findings confined to the population from which the sample was drawn?	X			
Ethics	18. Were participants asked to provide (informed) consent or assent?	X			
Ethics	19. Were participants debriefed at the end of data collection?		X		
Ethics	20. Were funding sources or conflicts of interest disclosed?	X			

SCORING (optional)

Overall Quality Score (%): 70%

Compute an overall study quality score expressed as a percentage by dividing YES (Y) scores by the Total (T) number of **APPLICABLE** items and multiplying by 100. If a report fails to attain a Y score for 5 of the items, then it may be classed as of questionable quality.
Specifically:
When (T) = 20, then a score of Y/T ≥ 75% may be considered ceptable quality.
When (T) = 19, then a score of Y/T ≥ 73% may be considered ceptable quality.
When (T) = 18, then a score of Y/T ≥ 72% may be considered ceptable quality.
When (T) = 17, then a score of Y/T ≥ 70% may be considered ceptable quality.
If Y/T <75% or < 73% or < 72%, or < 70% (depending on number of applicable items), then study is of questionable quality.

Domain Quality Scores
Express domain quality scores as a simple ratio of the (Y) items, divided by the (T) applicable items.

(4 items) Introduction (Rationale/Variables) score: /4

(3 items) Participants (Sampling/Recruitment) score: /3

(10 items) Data (Collection/Analyses/Measures/Results/Discussion) score: /10

(3 items) Ethics score: /3

IN A DATA FILE, ASSIGN **1** FOR YES SCORES; **0** FOR NO OR NOT STATED CLEARLY; AND **2** = FOR NOT APPLICABLE.

Quality Assessment Checklist for Survey Studies in Psychology (Q-SSP) and Guide

Study: Self-Efficacy as a Mechanism Linking Daily Stress to Mental Health in Students: A Three-Wave Cross-Lagged Study					
**Research domain**	**Quality item**	**Yes**	**No**	**Not stated clearly**	**N/A**
Introduction (Rationale)	1. Was the problem or phenomenon under investigation defined, described, and justified?	X			
Introduction (Rationale)	2. Was the population under investigation defined, described, and justified?	X			
Introduction (Rationale)	3. Were specific research questions and/or hypotheses stated?	X			
Introduction (Variables)	4. Were operational definitions of all study variables provided?	X			
Participants (Sampling)	5. Were participant inclusion criteria stated?	X			
Participants (Sampling)	6. Was the participant recruitment strategy described?			X	
Participants (Sampling)	7. Was a justification/ rationale for the sample size provided?		X		
Data (Collection)	8. Was the attrition rate provided? (applies to cross-sectional and prospective studies)		X		
Data (Analyses)	9. Was a method of treating attrition provided? (applies to cross-sectional and prospective studies)	X			
Data (Analyses)	10. Were the data analysis techniques justified (i.e., was the link between hypotheses/ aims / research questions and data analyses explained)?	X			
Data (Measures)	11. Were the measures provided in the report (or in a supplement) in full?		X		
Data (Measures)	12. Was evidence provided for the validity of all the measures (or instrument) used?	X			
Data (Collection)	13. Was information provided about the person(s) who collected the data (e.g., training, expertise, other demographic characteristics)?		X		
Data (Collection)	14. Was information provided about the context (e.g., place) of data collection?		X		
Data (Collection)	15. Was information provided about the duration (or start and end date) of data collection?		X		
Data (Results)	16. Was the study sample described in terms of key demographic characteristics?	X			

Data (Discussion)	17. Was discussion of findings confined to the population from which the sample was drawn?	X			
Ethics	18. Were participants asked to provide (informed) consent or assent?	X			
Ethics	19. Were participants debriefed at the end of data collection?		X		
Ethics	20. Were funding sources or conflicts of interest disclosed?	X			

SCORING (optional)

Overall Quality Score (%): 60%

Compute an overall study quality score expressed as a percentage by dividing YES (Y) scores by the Total (T) number of **APPLICABLE** items and multiplying by 100. If a report fails to attain a Y score for 5 of the items, then it may be classed as of questionable quality.

Specifically:

When (T) = 20, then a score of Y/T ≥ 75% may be considered ceptable quality.

When (T) = 19, then a score of Y/T ≥ 73% may be considered ceptable quality.

When (T) = 18, then a score of Y/T ≥ 72% may be considered ceptable quality.

When (T) = 17, then a score of Y/T ≥ 70% may be considered ceptable quality.

If Y/T <75% or < 73% or < 72%, or < 70% (depending on number of applicable items), then study is of questionable quality.

Domain Quality Scores

Express domain quality scores as a simple ratio of the (Y) items, divided by the (T) applicable items.

(4 items) Introduction (Rationale/Variables) score: /4

(3 items) Participants (Sampling/Recruitment) score: /3

(10 items) Data (Collection/Analyses/Measures/Results/Discussion) score: /10

(3 items) Ethics score: /3

IN A DATA FILE, ASSIGN **1** FOR YES SCORES; **0** FOR NO OR NOT STATED CLEARLY; AND **2** = FOR NOT APPLICABLE.

Quality Assessment Checklist for Survey Studies in Psychology (Q-SSP) and Guide

Study: Self-critical thinking mediates the relationship between perfectionism and perceived stress in undergraduate students: A longitudinal study

Research domain	Quality item	Yes	No	Not stated clearly	N/A
Introduction (Rationale)	1. Was the problem or phenomenon under investigation defined, described, and justified?	X			
Introduction (Rationale)	2. Was the population under investigation defined, described, and justified?	X			
Introduction (Rationale)	3. Were specific research questions and/or hypotheses stated?	X			
Introduction (Variables)	4. Were operational definitions of all study variables provided?	X			
Participants (Sampling)	5. Were participant inclusion criteria stated?	X			
Participants (Sampling)	6. Was the participant recruitment strategy described?	X			
Participants (Sampling)	7. Was a justification/ rationale for the sample size provided?	X			
Data (Collection)	8. Was the attrition rate provided? (applies to cross-sectional and prospective studies)	X			
Data (Analyses)	9. Was a method of treating attrition provided? (applies to cross-sectional and prospective studies)	X			
Data (Analyses)	10. Were the data analysis techniques justified (i.e., was the link between hypotheses/ aims / research questions and data analyses explained)?	X			
Data (Measures)	11. Were the measures provided in the report (or in a supplement) in full?		X		
Data (Measures)	12. Was evidence provided for the validity of all the measures (or instrument) used?	X			
Data (Collection)	13. Was information provided about the person(s) who collected the data (e.g., training, expertise, other demographic characteristics)?		X		
Data (Collection)	14. Was information provided about the context (e.g., place) of data collection?		X		
Data (Collection)	15. Was information provided about the duration (or start and end date) of data collection?		X		
Data (Results)	16. Was the study sample described in terms of key demographic characteristics?		X		

Data (Discussion)	17. Was discussion of findings confined to the population from which the sample was drawn?	X			
Ethics	18. Were participants asked to provide (informed) consent or assent?	X			
Ethics	19. Were participants debriefed at the end of data collection?			X	
Ethics	20. Were funding sources or conflicts of interest disclosed?	X			

SCORING (optional)

Overall Quality Score (%): 70%

Compute an overall study quality score expressed as a percentage by dividing YES (Y) scores by the Total (T) number of **APPLICABLE** items and multiplying by 100. If a report fails to attain a Y score for 5 of the items, then it may be classed as of questionable quality.
Specifically:
When (T) = 20, then a score of Y/T ≥ 75% may be considered ceptable quality.
When (T) = 19, then a score of Y/T ≥ 73% may be considered ceptable quality.
When (T) = 18, then a score of Y/T ≥ 72% may be considered ceptable quality.
When (T) = 17, then a score of Y/T ≥ 70% may be considered ceptable quality.
If Y/T <75% or < 73% or < 72%, or < 70% (depending on number of applicable items), then study is of questionable quality.

Domain Quality Scores
Express domain quality scores as a simple ratio of the (Y) items, divided by the (T) applicable items.

(4 items) Introduction (Rationale/Variables) score: /4

(3 items) Participants (Sampling/Recruitment) score: /3

(10 items) Data (Collection/Analyses/Measures/Results/Discussion) score: /10

(3 items) Ethics score: /3

IN A DATA FILE, ASSIGN **1** FOR YES SCORES; **0** FOR NO OR NOT STATED CLEARLY; AND **2** = FOR NOT APPLICABLE.

Quality Assessment Checklist for Survey Studies in Psychology (Q-SSP) and Guide

Study: Self-blame and stress in undergraduate college students: The mediating role of proactive coping

**Research domain**	**Quality item**	**Yes**	**No**	**Not stated clearly**	**N/A**
Introduction (Rationale)	1. Was the problem or phenomenon under investigation defined, described, and justified?	X			
Introduction (Rationale)	2. Was the population under investigation defined, described, and justified?			X	
Introduction (Rationale)	3. Were specific research questions and/or hypotheses stated?	X			
Introduction (Variables)	4. Were operational definitions of all study variables provided?	X			
Participants (Sampling)	5. Were participant inclusion criteria stated?	X			
Participants (Sampling)	6. Was the participant recruitment strategy described?	X			
Participants (Sampling)	7. Was a justification/ rationale for the sample size provided?		X		
Data (Collection)	8. Was the attrition rate provided? (applies to cross-sectional and prospective studies)		X		
Data (Analyses)	9. Was a method of treating attrition provided? (applies to cross-sectional and prospective studies)		X		
Data (Analyses)	10. Were the data analysis techniques justified (i.e., was the link between hypotheses/ aims / research questions and data analyses explained)?	X			
Data (Measures)	11. Were the measures provided in the report (or in a supplement) in full?		X		
Data (Measures)	12. Was evidence provided for the validity of all the measures (or instrument) used?	X			
Data (Collection)	13. Was information provided about the person(s) who collected the data (e.g., training, expertise, other demographic characteristics)?		X		
Data (Collection)	14. Was information provided about the context (e.g., place) of data collection?	X			
Data (Collection)	15. Was information provided about the duration (or start and end date) of data collection?	X			
Data (Results)	16. Was the study sample described in terms of key demographic characteristics?	X			

Data (Discussion)	17. Was discussion of findings confined to the population from which the sample was drawn?	X			
Ethics	18. Were participants asked to provide (informed) consent or assent?		X		
Ethics	19. Were participants debriefed at the end of data collection?		X		
Ethics	20. Were funding sources or conflicts of interest disclosed?		X		

SCORING (optional)

Overall Quality Score (%): 55

Compute an overall study quality score expressed as a percentage by dividing YES (Y) scores by the Total (T) number of **APPLICABLE** items and multiplying by 100. If a report fails to attain a Y score for 5 of the items, then it may be classed as of questionable quality.
Specifically:
When (T) = 20, then a score of Y/T ≥ 75% may be considered ceptable quality.
When (T) = 19, then a score of Y/T ≥ 73% may be considered ceptable quality.
When (T) = 18, then a score of Y/T ≥ 72% may be considered ceptable quality.
When (T) = 17, then a score of Y/T ≥ 70% may be considered ceptable quality.
If Y/T <75% or < 73% or < 72%, or < 70% (depending on number of applicable items), then study is of questionable quality.

Domain Quality Scores
Express domain quality scores as a simple ratio of the (Y) items, divided by the (T) applicable items.

(4 items) Introduction (Rationale/Variables) score: 3/4

(3 items) Participants (Sampling/Recruitment) score: 2/3

(10 items) Data (Collection/Analyses/Measures/Results/Discussion) score: 6/10

(3 items) Ethics score: 2/2

IN A DATA FILE, ASSIGN **1** FOR YES SCORES; **0** FOR NO OR NOT STATED CLEARLY; AND **2** = FOR NOT APPLICABLE.

Quality Assessment Checklist for Survey Studies in Psychology (Q-SSP) and Guide

Study: Sleep and Perceived Stress: An Exploratory Mediation Analysis of the Role of Self-Control and Resilience among University Students					
**Research domain**	**Quality item**	**Yes**	**No**	**Not stated clearly**	**N/A**
Introduction (Rationale)	1. Was the problem or phenomenon under investigation defined, described, and justified?	X			
Introduction (Rationale)	2. Was the population under investigation defined, described, and justified?	X			
Introduction (Rationale)	3. Were specific research questions and/or hypotheses stated?	X			
Introduction (Variables)	4. Were operational definitions of all study variables provided?	X			
Participants (Sampling)	5. Were participant inclusion criteria stated?	X			
Participants (Sampling)	6. Was the participant recruitment strategy described?	X			
Participants (Sampling)	7. Was a justification/ rationale for the sample size provided?	X			
Data (Collection)	8. Was the attrition rate provided? (applies to cross-sectional and prospective studies)	X			
Data (Analyses)	9. Was a method of treating attrition provided? (applies to cross-sectional and prospective studies)		X		
Data (Analyses)	10. Were the data analysis techniques justified (i.e., was the link between hypotheses/ aims / research questions and data analyses explained)?	X			
Data (Measures)	11. Were the measures provided in the report (or in a supplement) in full?	X			
Data (Measures)	12. Was evidence provided for the validity of all the measures (or instrument) used?	X			
Data (Collection)	13. Was information provided about the person(s) who collected the data (e.g., training, expertise, other demographic characteristics)?		X		
Data (Collection)	14. Was information provided about the context (e.g., place) of data collection?		X		
Data (Collection)	15. Was information provided about the duration (or start and end date) of data collection?		X		
Data (Results)	16. Was the study sample described in terms of key demographic characteristics?		X		

Data (Discussion)	17. Was discussion of findings confined to the population from which the sample was drawn?	X			
Ethics	18. Were participants asked to provide (informed) consent or assent?	X			
Ethics	19. Were participants debriefed at the end of data collection?		X		
Ethics	20. Were funding sources or conflicts of interest disclosed?	X			

SCORING (optional)

Overall Quality Score (%): 70%

Compute an overall study quality score expressed as a percentage by dividing YES (Y) scores by the Total (T) number of **APPLICABLE** items and multiplying by 100. If a report fails to attain a Y score for 5 of the items, then it may be classed as of questionable quality.

Specifically:

When (T) = 20, then a score of Y/T ≥ 75% may be considered ceptable quality.

When (T) = 19, then a score of Y/T ≥ 73% may be considered ceptable quality.

When (T) = 18, then a score of Y/T ≥ 72% may be considered ceptable quality.

When (T) = 17, then a score of Y/T ≥ 70% may be considered ceptable quality.

If Y/T <75% or < 73% or < 72%, or < 70% (depending on number of applicable items), then study is of questionable quality.

Domain Quality Scores

Express domain quality scores as a simple ratio of the (Y) items, divided by the (T) applicable items.

(4 items) Introduction (Rationale/Variables) score: /4

(3 items) Participants (Sampling/Recruitment) score: /3

(10 items) Data (Collection/Analyses/Measures/Results/Discussion) score: /10

(3 items) Ethics score: /3

IN A DATA FILE, ASSIGN **1** FOR YES SCORES; **0** FOR NO OR NOT STATED CLEARLY; AND **2** = FOR NOT APPLICABLE.

Quality Assessment Checklist for Survey Studies in Psychology (Q-SSP) and Guide

Study: Sex matters: stress perception and the relevance of resilience and perceived social support in emerging adults

Research domain	Quality item	Yes	No	Not stated clearly	N/A
Introduction (Rationale)	1. Was the problem or phenomenon under investigation defined, described, and justified?	X			
Introduction (Rationale)	2. Was the population under investigation defined, described, and justified?	X			
Introduction (Rationale)	3. Were specific research questions and/or hypotheses stated?	X			
Introduction (Variables)	4. Were operational definitions of all study variables provided?		X		
Participants (Sampling)	5. Were participant inclusion criteria stated?	X			
Participants (Sampling)	6. Was the participant recruitment strategy described?		X		
Participants (Sampling)	7. Was a justification/ rationale for the sample size provided?		X		
Data (Collection)	8. Was the attrition rate provided? (applies to cross-sectional and prospective studies)		X		
Data (Analyses)	9. Was a method of treating attrition provided? (applies to cross-sectional and prospective studies)		X		
Data (Analyses)	10. Were the data analysis techniques justified (i.e., was the link between hypotheses/ aims / research questions and data analyses explained)?	X			
Data (Measures)	11. Were the measures provided in the report (or in a supplement) in full?		X		
Data (Measures)	12. Was evidence provided for the validity of all the measures (or instrument) used?	X			
Data (Collection)	13. Was information provided about the person(s) who collected the data (e.g., training, expertise, other demographic characteristics)?		X		
Data (Collection)	14. Was information provided about the context (e.g., place) of data collection?		X		
Data (Collection)	15. Was information provided about the duration (or start and end date) of data collection?		X		
Data (Results)	16. Was the study sample described in terms of key demographic characteristics?	X			

Data (Discussion)	17. Was discussion of findings confined to the population from which the sample was drawn?	X			
Ethics	18. Were participants asked to provide (informed) consent or assent?		X		
Ethics	19. Were participants debriefed at the end of data collection?			X	
Ethics	20. Were funding sources or conflicts of interest disclosed?	X			

SCORING (optional)

Overall Quality Score (%): 45%

Compute an overall study quality score expressed as a percentage by dividing YES (Y) scores by the Total (T) number of **APPLICABLE** items and multiplying by 100. If a report fails to attain a Y score for 5 of the items, then it may be classed as of questionable quality.
Specifically:
When (T) = 20, then a score of Y/T ≥ 75% may be considered ceptable quality.
When (T) = 19, then a score of Y/T ≥ 73% may be considered ceptable quality.
When (T) = 18, then a score of Y/T ≥ 72% may be considered ceptable quality.
When (T) = 17, then a score of Y/T ≥ 70% may be considered ceptable quality.
If Y/T <75% or < 73% or < 72%, or < 70% (depending on number of applicable items), then study is of questionable quality.

Domain Quality Scores
Express domain quality scores as a simple ratio of the (Y) items, divided by the (T) applicable items.

(4 items) Introduction (Rationale/Variables) score: /4

(3 items) Participants (Sampling/Recruitment) score: /3

(10 items) Data (Collection/Analyses/Measures/Results/Discussion) score: /10

(3 items) Ethics score: /3

IN A DATA FILE, ASSIGN **1** FOR YES SCORES; **0** FOR NO OR NOT STATED CLEARLY; AND **2** = FOR NOT APPLICABLE.
